# Diaphragm thickness modifications and associated factors during VA-ECMO for a cardiogenic shock: a cohort study

**DOI:** 10.1186/s13613-024-01264-8

**Published:** 2024-03-08

**Authors:** Pierre-Henri Moury, Alexandre Béhouche, Sébastien Bailly, Zoé Durand, Géraldine Dessertaine, Angelina Pollet, Samir Jaber, Samuel Verges, Pierre Albaladejo

**Affiliations:** 1 Pôle Anesthésie-Réanimation, Grenoble Alpes University, Grenoble, France; 2 Univ. Grenoble Alpes, Inserm, Grenoble Alpes University Hospital, HP2 Laboratory, Grenoble, France; 3 Intensive Care Unit, Anaesthesiology and Critical Care Department B, Saint Eloi Teaching Hospital, Université Montpellier 1, Centre Hospitalier Universitaire Montpellier, Montpellier, France

**Keywords:** Diaphragm, Muscle, Weaning, ECMO, Cardiogenic shock

## Abstract

**Background:**

The incidence, causes and impact of diaphragm thickness evolution in veno-arterial extracorporeal membrane oxygenation (VA-ECMO) for cardiogenic shock are unknown.

Our study investigates its evolution during the first week of VA-ECMO and its relationship with sweep gas flow settings.

**Methods:**

We conducted a prospective monocentric observational study in a 12-bed ICU in France, enrolling patients on the day of the VA-ECMO implantation. The diaphragm thickness and the diaphragm thickening fraction (as index of contractile activity, dTF; dTF < 20% defined a low contractile activity) were daily measured for one week using ultrasound. Factors associated with diaphragm thickness evolution (categorized as increased, stable, or atrophic based on > 10% modification from baseline to the last measurement), early extubation role (< day4), and patients outcome at 60 days were investigated. Changes in diaphragm thickness, the primary endpoint, was analysed using a mixed-effect linear model (MLM).

**Results:**

Of the 29 included patients, seven (23%) presented diaphragm atrophy, 18 remained stable (60%) and 4 exhibited an increase (17%). None of the 13 early-extubated patients experienced diaphragm atrophy, while 7 (46%) presented a decrease when extubated later (p-value = 0.008). Diaphragm thickness changes were not associated with the dTF (p-value = 0.13) but with sweep gas flow (Beta = − 3; Confidence Interval at 95% (CI) [− 4.8; − 1.2]. p-value = 0.001) and pH (Beta = − 2; CI [− 2.9; − 1]. p-value < 0.001) in MLM. The dTF remained low (< 20%) in 20 patients (69%) at the study’s end and was associated with sweep gas flow evolution in MLM (Beta = − 2.8; 95% CI [− 5.2; − 0.5], p-value = 0.017). Odds ratio of death at 60 days in case of diaphragm atrophy by day 7 was 8.50 ([1.4–74], p = 0.029).

**Conclusion:**

In our study, diaphragm thickness evolution was frequent and not associated with the diaphragm thickening fraction. Diaphragm was preserved from atrophy in case of early extubation with ongoing VA-ECMO assistance. Metabolic disorders resulting from organ failures and sweep gas flow were linked with diaphragm thickness evolution. Preserved diaphragm thickness in VA-ECMO survivors emphasizes the importance of diaphragm-protective strategies, including meticulous sweep gas flow titration.

**Supplementary Information:**

The online version contains supplementary material available at 10.1186/s13613-024-01264-8.

## Key points

**Question:**

How does the diaphragm thickness evolves during the first week of VA-ECMO treatment for a cardiogenic shock and is it associated with sweep gas flow settings?

**Findings:**

In patient receiving VA-ECMO for a cardiogenic shock, fewer progressive atrophy was described when early extubation was possible and associated with lower death rate at two months. The diaphragm thickness increased with the sweep gas flow and decreased with the acidosis. Diaphragm thickness evolution did not evolve parallel to the diaphragm thickening fraction.

**Meaning:**

In VA-ECMO-treated patients, we advocate for diaphragmatic-protective strategies through the careful titration of sweep gas flows, tight pH control, and the promotion of early extubation.

**Supplementary Information:**

The online version contains supplementary material available at 10.1186/s13613-024-01264-8.

## Introduction

Cardiogenic shock often necessitates mechanical ventilation due to accompanying acute respiratory failure [1, 2]. Heart failure has been demonstrated to be linked to diaphragm weakness in non-critically ill patients [3–7]. The diaphragm, as the primary respiratory muscle, plays a vital role in facilitating liberation from mechanical ventilation [8, 9]. Diaphragm weakness acquired during the intensive care unit (ICU) stay have been reported in the literature [10, 11]. The prevalence of diaphragm weakness can vary from 64% of the patient after 24 h of intubation to 80% at the time of weaning [8, 12]. Various factors have been identified as potential causes, including sepsis, mechanical ventilation, cardiac surgery, and shock [13, 14]. Furthermore, acquired diaphragm weakness may be associated with mortality and difficult weaning from mechanical ventilation [13, 15, 16].

In the ICU, circulatory failures often lead to veno-arterial extracorporeal membrane oxygenation (VA-ECMO). Therefore, several factors introduce uncertainties in the relationship between cardiogenic shock and acquired diaphragm dysfunction. While animal studies have reported diaphragm weakness in experimental cardiogenic shock, limited documentation exists in humans [17, 18]. Our recent findings indicated that changes in VA-ECMO sweep gas flow significantly influenced diaphragm thickening fraction (dTF), serving as a surrogate for diaphragm effort measurement [18]. Other studies have shown that veno-venous extracorporeal membrane oxygenation (VV-ECMO) greatly facilitate in achieving lung and diaphragm protective ventilation targets [18, 19]. Recent findings have also established a relationship between diaphragm thickness evolution in mechanically ventilated patients and diaphragm contractile activity [20]. Consequently, it has been hypothesized that carefully titrating respiratory support could prevent structural impairments of the diaphragm.

Therefore, our primary objective was to investigate changes in diaphragm thickness during the first week of VA-ECMO treatment for cardiogenic shock. Secondary goals included exploring associated factors such as VA-ECMO settings, early extubation, organ failure, pharmacological drug use, sepsis, and a history of cardiac surgery. Finally, given the rate of exposition to mechanical ventilation, and the role of sweep gas flow on diaphragm contractility in our previous study [18], we hypothesized that distinct diaphragm thickness evolution patterns can be delineated and potentially linked to sweep gas flow settings during VA-ECMO treatment.

## Methods

### Design

This physiological prospective observational study, approved on 16th of July 2019, by the Comité de Protection des Personnes Ile de France V (“AtrophyECMO”: IRB number 2019-A01273-54), was registered at ClinicalTrial.gov (NCT04052230). Conducted from October 2019 to February 2022, it adhered to the Helsinki Declaration of 1975. The need for written consent was waived according to French Law. Oral consent was obtained from each patient or from a close relative.

### Patients

Patients were consecutively enrolled in the study if they were treated with a peripheral VA-ECMO for cardiogenic shock. Exclusion criteria were VV-ECMO and the inability to perform diaphragm ultrasound, including patients in moribund state.

Demographic data, severity scores, comorbidities, reasons for admission to the ICU and for initiating VA-ECMO were collected. The mechanical ventilation duration, duration of VA-ECMO, the bridge to a left ventricle assistance device or a heart transplantation, duration of ICU stay and ICU mortality were also collected. Arterial blood gases were drawn from the right upper extremity.

Mechanical ventilation, sedation, ECMO care, and patient outcome are described in the Additional file 1 data materials. Briefly, Mechanical ventilation was initially set to control modes before pressure support ventilation to insure a tidal volume below the target of 6 ml/kg of predicted ideal body weight and < 14 cmH_2_O driving pressure threshold. A VA-ECMO blender was systematically used to set the sweep gas flow and VA-ECMO membrane Oxygen fraction in intubated and extubated patient. A lower sweep gas flow threshold of 1.5 L/min was respected to avoid hypoxemia due to the veno-arterial shunt. The VA-ECMO sweep gas flow was set to provide an appropriate pH and PaCO_2_ balance according to the tidal volume target and the driving pressure target.

We assessed the Richmond Agitation Sedation Scale (RASS). Sedation, vasoactive agents, and corticoids and neuromuscular blocking agent used were collected daily.

### Diaphragm assessment and study procedure

On the first day of VA-ECMO assistance, measurements of the thickness of the right hemidiaphragm were done at the end of inspiration (T_EI_, mm) and expiration (T_EE_, mm) [21]. Ultrasound measurements were conducted independently through a computer-driven software on images extracted from the data base (DICOM viewer 3.0, Philips, Netherlands). Ultrasounds measurement of the diaphragm thickness, thresholds of thickness evolution and thickening have been extensively reported and described in previous studies [8, 14, 18, 22]. The diaphragm thickening fraction (dTF) was calculated as (T_EI_-T_EE_)/T_EE_ and expressed as percentage. Ultrasound measurements were recorded daily, measured and averaged over at least 3 respiratory cycles. Ultrasound measurements were conducted by three intensivist (AB, ZD, PHM) trained in diaphragm imaging [8, 14, 18]. Diaphragm thickness was measured systematically in a 20–30° upright position in the zone of apposition of the diaphragm with the liver between the 8th–10th intercostal spaces using a 7.5–12 MHz probe [23, 24]. The reproducibility was reported in previous study from the team and was not investigated in the presented study [8, 14, 18]. The place of the probe was systematically marked. Ideally, the ultrasound measurements were undertaken daily whether the patient were intubated and ventilated in control modes or spontaneously breathing. Ideally, we standardized the daily spontaneous breathing trial which was sat to 0 cmH_2_O of PEEP level and 7 cmH_2_O of pressure support ventilation if considered as possible. The ventilator settings and the use of sedatives or neuromuscular blocking agents within the last 24 h were noted during the assessment. Physiological variables, blood gas, and an ultrasound measurement of the left ventricle ejection fraction (LVEF) were collected at the same time [25, 26]. The T_EE_ evolution was categorized in three groups from the baseline to the last ultrasound assessment obtained during the first week: decreased by 10%, increased by 10%, or stability [20]. In unplanned analysis, T_EE_ evolution was also categorized in two groups atrophy and non-atrophy (according to the presence or absence of a 10% decrease in thickness). During the same ultrasound assessment, we used the 20% dTF threshold to define a low diaphragm contractile activity [8, 14].

### Endpoints

The primary endpoint was to assess the T_EE_ evolution during the first week of VA-ECMO. The secondary endpoints were the assessment of predefined influential factors on T_EE_ evolution during the first week, and dTF evolution.

These parameters were: low diaphragm contractility, SOFA score, SAPS2 score, settings of mechanical ventilation and VA-ECMO, sedation, pharmacological drugs use, kidney function and the association with the LVEF.

Lastly, the patient outcome were analysed, including: early weaning from mechanical ventilation impact (before day 4), mechanical ventilation duration, and mortality at 60 days.

### Statistical analyses

Statistical analyses were performed using R software. Categorical variables were expressed as numbers (percentages) and if the assumptions for the Chi-squared test were not fulfilled, the Fisher exact test was applied. Continuous variables were expressed as median (interquartile range [25, 75%]) and the Shapiro test was used to test normality. Comparisons were made between patients who presented diaphragm atrophy or not using Student’s t-test or Mann–Whitney test for parametric and non-parametric variables, respectively.

In descriptive analysis, given the longitudinal follow-up design, we used two strategies to characterise the variable before conducting an analytic model construction: definition of categories of diaphragm evolution and repeated measures analysis [20]. We reported the average values of the first three days of the potential risk factors among predefined categories. We used repeated measure correlation (RMCr, 95% confidence intervals or CI) to evaluate correlations [27]. Then, the primary analysis was the calculation of a mixed-effect linear model (MLM) of diaphragm thickness over time, with subjects treated as random effects [28].

Three categories were defined whether the T_EE_ had decreased by 10%, increased by 10%, or remained stable [20]. Using the Bonferroni correction and Dunn’s test, we divided the population into two subgroups based on whether the diaphragm thickness had decreased by 10% or not, creating an atrophy group and a no-atrophy group.

The MLM was chosen for primary analysis because of its efficiency to incorporate repeated measures and to handle missing values [28]. Logit transformations were used to normalize values included in the MLM to improve the stability of the model [28]. Then, sensitivity analysis were performed to corroborate our model (see Additional file 1). In secondary analysis, we also used a MLM to characterize influential factors of dTF.

Patients’ outcomes at 60 days was studied by a univariable analysis in case of atrophy or not. Secondly, we conducted a logistic regression to calculate the odds ratio (OR) of death at 60 days.

A *p-* value of less than 0.05 was considered statistically significant.

Sample size calculation was performed based on preliminary clinical results and described in the Additional file 1 data materials.

## Results

### Patient characteristics

**Fig. 1:**
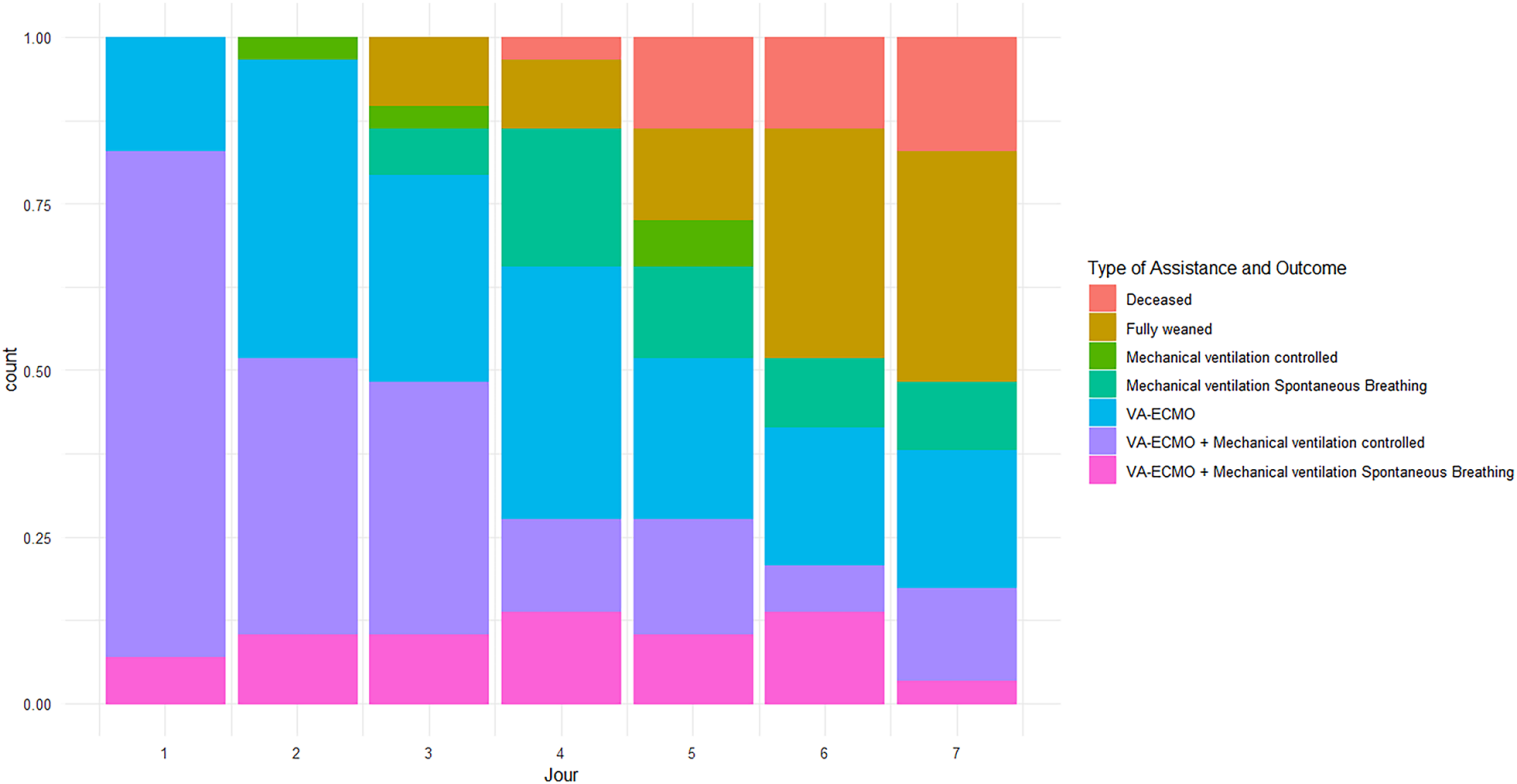
Patients requiring assistances. The histogram illustrates the cumulative rate of patients’ specific assistances or death over the study period in days. Patients were categorized based on the level of assistance, including full assistance with both VA-ECMO and mechanical ventilation in either control or spontaneous breathing modes. Additionally, patients assisted with VA-ECMO alone were included, and those followed after removal from assistance. Patients were also classified based on their respiratory status, which included being assisted by mechanical ventilation (either in control or spontaneous breathing modes) or being fully weaned from the need for intubation and VA-ECMO

During the study period, spanning 18 months due to service reorganization amid the COVID-19 pandemic, a trained ultrasound operator was intermittently present for ten, three, and five months, respectively. Among the 61 patients implanted with an ECMO during this period, 10 were moribund at admission, 5 had a VV-ECMO and six were admitted during the absence of the trained operator (see Additional file 1: Figure S1). A total of thirty patients were initially considered, but one significant outlier was excluded due to poor image quality, resulting in 29 patients for analysis with a total of 135 T_EE_ assessments. Five patients (17%) died before day seven, and ten (34%) died before day sixty. Two patients underwent heart transplantation, and one had a left ventricle assistance device procedure (Additional file 1: Table S1). None of the patients had a combined assistance by Impella® or Intra-aortic balloon pump during VA-ECMO. Ventilation was initially provided in control modes, transitioning to spontaneous breathing before potential extubation, irrespective continued of VA-ECMO (Fig. 1). At the end of the first inclusion day, two patients were never intubated, and four were extubated, precluding diaphragm thickness measurement while intubated during their study period. Thirteen patients (45%) remained intubated on day 3 (see Additional file 1: Table S2). Patients were ventilated for six days [1, 11], and VA-ECMO assistance lasted six days [3, 9]. Dialysis requirement and acute kidney injury according to the KDIGO classification (Kidney Disease Improving Global Outcomes) at the end of the first week were not different according the T_EE_ classification groups (Table 1).

**Table 1:** Patients’ characteristics

Variable	Patient characteristics according to three groups of diaphragm thickness evolution (: stable, atrophy, increase) using a ultrasound method				Patient characteristics according to the absence or the presence of diaphragm atrophy using a ultrasound method		
	Increase, N = 4^1^	Stable, N = 18^1^	Atrophy, N = 7^1^	p-value^2^	Non-atrophy, N = 22^1^	Atrophy, N = 7^1^	p-value^3^
Age (years)	67 [66, 68]	52 [48, 65]	55 [47, 66]	0.2	62 [48, 67]	55 [47, 66]	0.8
Sex (male)	4 [100%]	15 [83%]	6 [86%]	> 0.9	19 [86%]	6 [86%]	> 0.9
Weight (kg)	90 [83, 93]	80 [64, 98]	85 [73, 86]	0.7	81 [66, 96]	85 [73, 86]	> 0.9
Height (m)	1.79 [1.73, 1.84]	1.75 [1.7, 1.8]	1.7 [1.7, 1.75]	0.5	1.75 [1.7, 1.8]	1.7 [1.7, 1.75]	0.4
Body Mass Index	28 [25, 29]	26 [23, 30]	26 [25, 28]	0.9	26 [23, 29]	26 [25, 28]	0.8
SOFA	9 [7, 11]	9 [6, 10]	9 [8, 16]	0.5	9 [6, 10]	9 [8, 16]	0.3
Severe Acute Physiology Score II	47 [45, 49]	43 [36, 51]	47 [40, 62]	0.6	44 [41, 51]	47 [40, 62]	0.6
Cardiac arrest	0 (0%)	9 (50%)	3 (43%)	0.3	9 (41%)	3 (43%)	> 0.9
Cardiac surgery	2 (50%)	6 (33%)	4 (57%)	0.5	8 (36%)	4 (57%)	0.4
COPD	1 (25%)	1 (5.6%)	0 (0%)	0.3	2 (9.1%)	0 (0%)	> 0.9
Cancer	1 (25%)	1 (5.6%)	0 (0%)	0.3	2 (9.1%)	0 (0%)	> 0.9
Chronic kidney insufficiency	1 (25%)	0 (0%)	0 (0%)	0.14	1 (4.5%)	0 (0%)	> 0.9
Dialysis	1 (25%)	2 (11%)	2 (29%)	0.5	3 (14%)	2 (29%)	0.6
Fluid balance at inclusion (ml)	− 346 [− 718, 178]	− 273 [-900, 486]	304 [− 936, 1,227]	0.9	− 273 [− 900, 486]	304 [− 936, 1,227]	0.7
Weight balance J0 to last assessment (kg)	4 [2, 4]	0 [-4, 2]	− 1 [− 7, 3]	0.4	1 [− 3, 4]	− 1 [− 7, 3]	0.6
KDIGO							
I	2 (50%)	13 (72%)	3 (43%)	0.4	15 (68%)	3 (43%)	0.4
II	0 (0%)	1 (5.6%)	0 (0%)		1 (4.5%)	0 (0%)	
III	2 (50%)	4 (22%)	4 (57%)		6 (27%)	4 (57%)	
Sepsis	0 (0%)	1 (5.6%)	1 (14%)	0.6	1 (4.5%)	1 (14%)	0.4
Number of MV days at day 7	2 [1, 4]	2 [1, 5]	6 [5, 7]	0.034	2 [1, 5]	6 [5, 7]	0.011
VA-ECMO parameters (All following parameters were averaged over the three first days)							
VA-ECMO Flow (l/min)	2.9 [2.9, 3]	3.5 [2, 4]	4.5 [3, 5]	0.3	3.2 [2.5, 3.9]	4.5 [3, 5.3]	0.2
Sweep gas flow (l/min)	3 [2.7, 3.2]	3.1 [2.6, 4]	4.8 [3, 5]	0.12	3 [2.7, 4]	4.8 [3.3, 5]	0.045

Results are reported for patients according to their diaphragm evolution status at the end of patient follow-up. We divided the study population in three categories whether the diaphragm thickness presented a ≥ 10% decrease, increase or a stability from baseline to the last assessment

^1^Median [interquartile range ]; n (%); ^2^Kruskal-Wallis rank sum test; Fisher’s exact test; ^3^Wilcoxon rank sum test; Fisher’s exact test; Wilcoxon rank sum exact test; *BMI*: Body mass index; *COPD:* Chronic Obstructive Pulmonary Disease; *SAPS2:* Simplified Acute Physiology Score 2*;MV:* Mechanical Ventilation; *VA-ECMO:* Veno-arterial extracorporeal membrane oxygenation; *KDIGO*: Kidney Disease Improving Global

### Diaphragm thickness

**Fig. 2:**
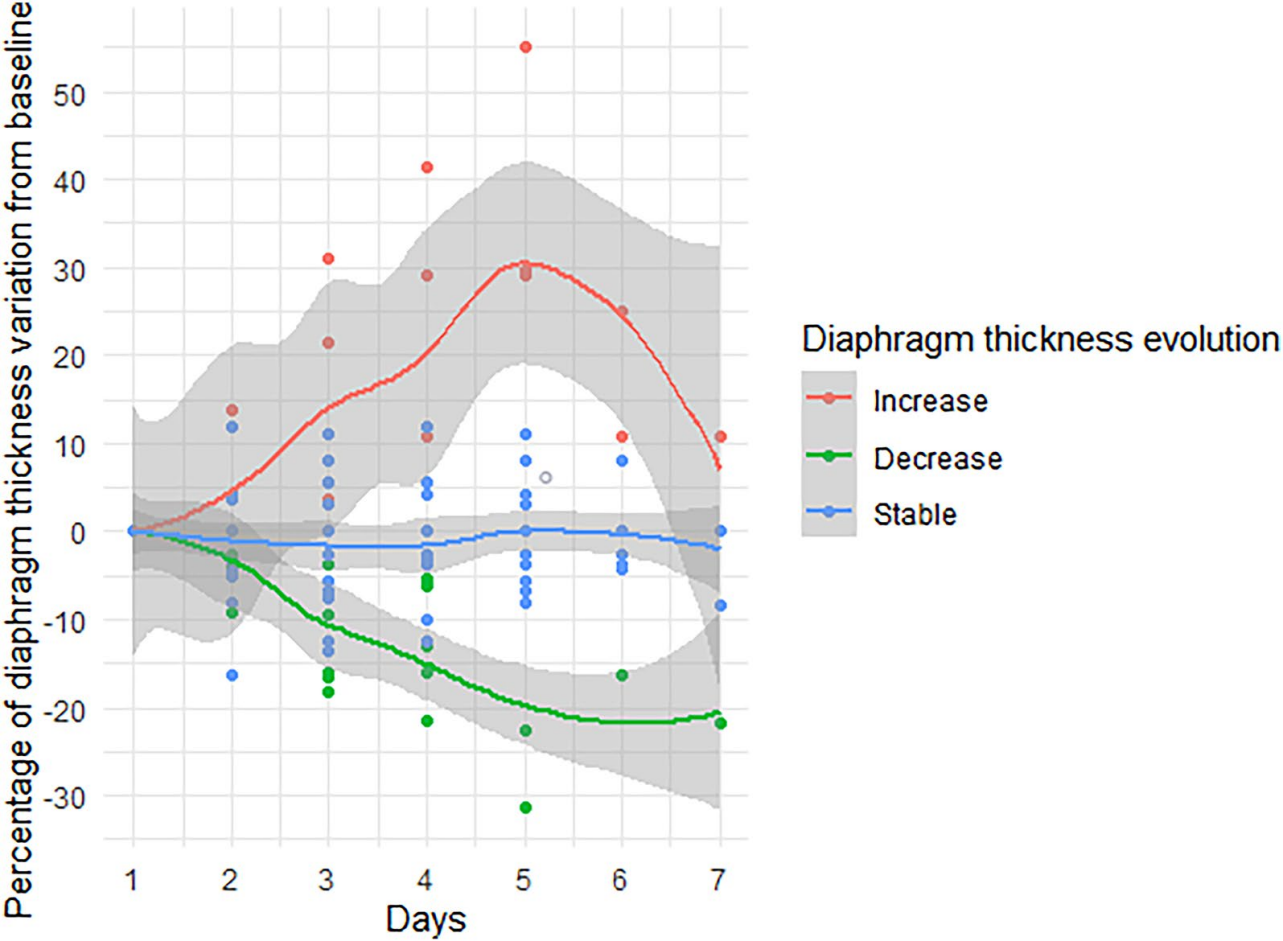
Evolution of diaphragm thickness from baseline until day 7. The panel shows the daily diaphragm thickness evolution from baseline in patients with diaphragm atrophy (green: decrease of 10% from baseline), increase (red: increase of 10% from baseline) and stability (blue) at the end of follow-up and expressed by percentage from baseline

**Fig. 3:**
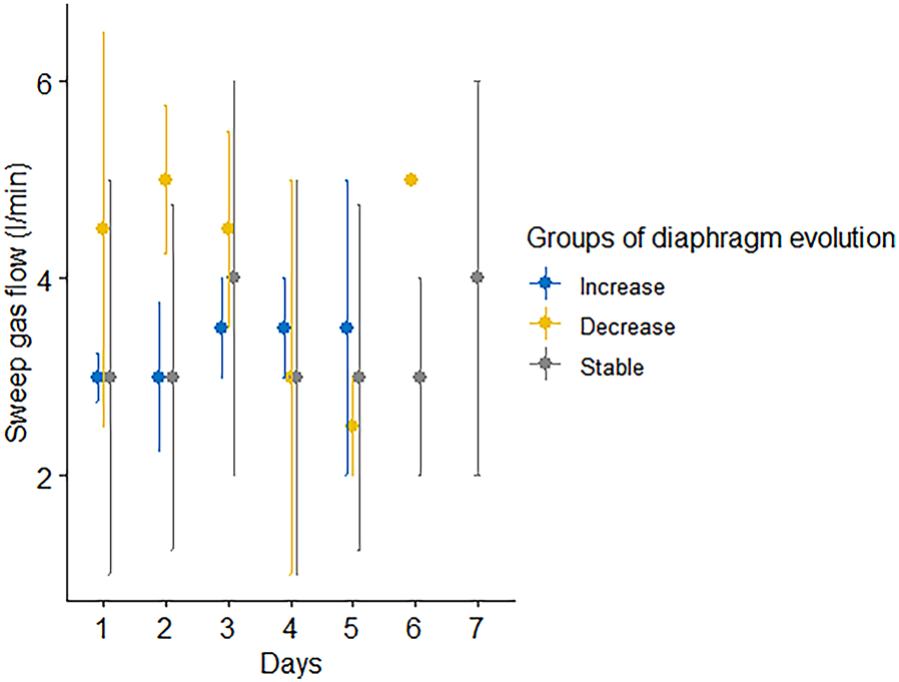
VA-ECMO sweep gas flow according the diaphragm thickness evolution at the end of follow-up. See Day 2 sweep gas flow was significantly different between groups (Kruskal–Wallis test, p-value = 0.01464, Fig. 3) and also when comparing the atrophy group to the stable group using Dunn’s test and Bonferroni correction (5 l/min [4, 5] to 3 l/min [2,4], p-value = 0.0066)

Individual values of T_EE_ from baseline are displayed in Additional file 1: Figure S2. The T_EE_ decrease was observed in 7 (23%) patients, remained stable in 18 patients (60%) and increased in 4 (17%) (Fig. 2). Among studied variables, sweep gas flow (Fig. 3), norepinephrine, neuromuscular blocking agent, and duration of mechanical ventilation differed among T_EE_ groups (Additional file 1: Table S3). Levosimendan was not administered to any patient before day 7.

On day 2, sweep gas flow was significantly different between groups (Kruskal–Wallis test, p-value = 0.01464, Fig. 3) and remained significant when comparing the atrophy group to the stable group using Dunn test and Bonferroni correction (5 l/min [4, 5] to 3 l/min [2,4], p-value = 0.0066).

Using repeated measures correlation (RMCr) to assess the relationship between daily variable changes and T_EE_ evolution, three parameters were significantly correlated: sweep gas flow (RMCr = 0.23, 95% CI: [0.01; 0.43], p-value = 0.039), daily insulin dose (RMCr = 0.32, 95% CI: [0.14; 0.49], p-value = 0.001), and pH (RMCr = − 0.2, 95% CI: [− 0.38; − 0.01], p-value = 0.038; Additional file 1: Figure S3). Subsequently, based on parsimony principles, a MLM was constructed, wherein the prominent factors were pH (Beta = − 2; 95% CI [− 2.9, − 1.1]. p-value < 0.001), sweep gas flow (Beta = − 3; 95% CI [− 4.8, − 1.2]. p-value = 0.001), and the interaction between pH and sweep gas flow (Beta = 0.41; 95% CI [0.17; 0.65]. p-value < 0.001), with no significant association between dTF and T_EE_ evolution (Additional file 1: Table S4). The T_EE_ evolution was not correlated with the dTF (RMCr = 0.25, 95% CI: [− 0.1; 0.54], p-value = 0.13), even after adjusting for confounding factors in a MLM. Daily fluid balance was not associated with T_EE_ evolution using repeated measure correlation and the MLM (RMCr = 0.03, 95% CI: [− 0.2; 0.2], p-value = 0.8). These results were confirmed in sensitivity analysis (see Additional file 1: Table S4).

### Diaphragm contractile activity

**Fig. 4:**
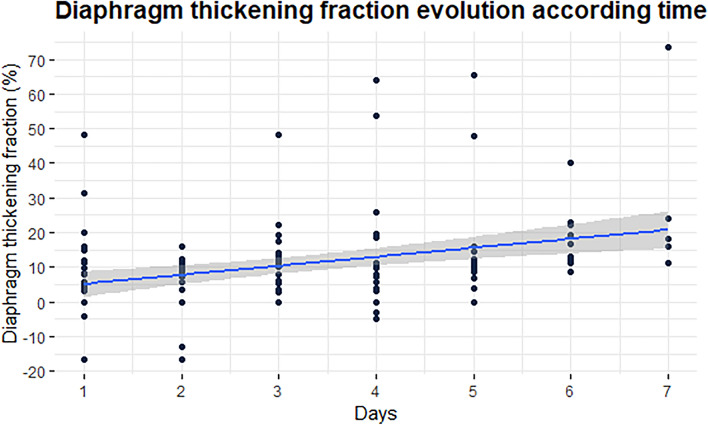
Diaphragm thickening fraction (dTF) evolution during the first week after implantation of a VA-ECMO. The panel shows the daily diaphragm thickening fraction (dTF) evolution from baseline to the end of follow-up and expressed by percentage from baseline

The diaphragm contractility, assessed by the dTF, increased throughout the study period from 5% [0, 13] on day one to 21% [17, 24] in six patients by day seven (Fig. 4). Although extubated patients had a higher dTF than intubated patients, it consistently remained below 20% until day 7 (see Additional file 1: Table S5). Twenty patients (69%) presented a dTF < 20% as surrogate for low diaphragm contractility at the end of the study period. The characteristics of the patients according to a 20% dTF threshold are described in the Additional file 1: Table S6. No correlation was found when comparing the sweep gas flow to the dTF using repeated measure correlation (RMCr = − 0.07, 95% CI: [− 0.29; 0.15], p-value = 0.54). However, analysis through MLM revealed an association between dTF and sweep gas flow (Beta = − 2.8; 95% CI [− 5.2; − 0.5], p-value = 0.017), as detailed in Additional file 1: Table S7.

### Outcome

Patients’ outcomes are detailed in Table 2. Notably, none of the patients liberated from mechanical ventilation before day 4 experienced diaphragm atrophy (Additional file 1: Table S8). Early extubation before day 4 was associated with significant alkalosis (p-value = 0.006), with lower sweep gas flow and PaCO_2_ (p-value = 0.002 and p-value = 0.003), with no differences in PaO_2_ or VA-ECMO flow (refer to Additional file 1: Figures S4-S7). Low diaphragm contractile did not exhibit an association with adverse outcomes (Additional file 1: Table S9).

**Table 2:** Patients outcomes in patients with or without diaphragm atrophy

Variable	Patient outcomes according to three groups of diaphragm thickness evolution (: stable, atrophy, increase) using a ultrasound method				Patient outcomes according to the absence or the presence of diaphragm atrophy using a ultrasound method		
	Increase, N = 4^1^	Decrease, N = 7^1^	Stable, N = 18^1^	p-value^2^	Non-atrophy, N = 22^1^	Atrophy, N = 7^1^	p-value^3^
Deceased before VA-ECMO weaning	1 (25%)	3 (43%)	4 (22%)	0.7	5 (23%)	3 (43%)	0.4
VA-ECMO weaning failure	1 (25%)	4 (57%)	7 (39%)	0.6	8 (36%)	4 (57%)	0.4
VA-ECMO days	5 [3.8, 7]	4 [2, 6]	6.5 [4, 11]	0.2	6 [4, 10]	4 [2, 6]	0.10
Days with mechanical ventilation	4 [2, 5]	8 [7, 20]	5 [1, 11]	0.059	5 [1, 10]	8 [7, 20]	0.029
Ventilator free days at day 60	55 [40, 58]	7 [0, 29]	54 [20, 59]	0.056	54 [20, 59]	7 [0, 29]	0.018
Deceased at day 60	1 (25%)	5 (71%)	4 (22%)	0.063	5 (23%)	5 (71%)	0.030
Deceased at day 7	1 (25%)	3 (43%)	1 (5.6%)	0.059	2 (9.1%)	3 (43%)	0.075

Results are reported for patients according to their diaphragm evolution status at the end of patient follow up. The population was divided in three categories whether the diaphragm thickness presented a 10% decrease, increase or remained stable from baseline to the last assessment. Outcomes are presented after 60 days of follow-up (unless otherwise specified)

^1^n (%); Median [interquartile range]

^2^Fisher’s exact test; Kruskal–Wallis rank sum test

^3^Fisher’s exact test; Wilcoxon rank sum test

Strikingly, none of the patient extubated before day 4 deceased within 60 days (p-value < 0.001). The OR of death at 60 days (Additional file 1: Table S10) for patients who experienced atrophy by day seven was 8.50 (95% CI [1.39; 74.09], p-value = 0.029). The model was adjusted for age, body mass index, cardiac arrest, SAPS2, the extubation at day 3 status and the presence of diaphragm atrophy by day 7. Further analysis of the patient deceased during this two months period is described in the Additional file 1: Table S11.

## Discussion

In a cohort of patients undergoing VA-ECMO for a cardiogenic shock, we observed frequent changes in diaphragm thickness (T_EE_), with a potential significant impact of an early extubation strategy in preventing myotrauma. Our study revealed that higher sweep gas flows and deeper acidosis were associated with increased diaphragm thickness. Notably, we described in our study that patients were preserved from diaphragm atrophy while a low early diaphragm contractile activity was maintained. Moreover, the presence of diaphragm atrophy was linked to higher mortality in this patient population.

These findings raise important concerns regarding the safeguarding diaphragm thickness changes during VA-ECMO assistance, highlighting the importance of early extubation and careful titration of sweep gas flow and pH control.

### Diaphragm thickness evolution during VA-ECMO

Diaphragm thickness changes in ICU patients receiving mechanical ventilation have been recently studied, with reported occurrences of disuse atrophy or increased thickness in critically ill individuals [10, 11, 29]. In our study, we described the dynamics of thickness changes during cardiogenic shock with VA-ECMO, influenced by factors such as mechanical ventilation exposure, sweep gas flow and metabolic disorders.

The decrease in T_EE_ in patients was observed in 23% of the population, and this decline was more prevalent in individuals experiencing adverse outcomes. While comparing the prevalence in our study to those conducted in ICU populations assisted by mechanical ventilation is challenging, the exposure to the primary underlying mechanism, mechanical ventilation, and its association with adverse outcomes align with established studies [20, 22].

Although we noted increased T_EE_ in some patients, the clinical significance of this was limited due to the small number of cases and the use of a debatable 10% increase threshold [21]. Notably, we found no specific correlation with causes of edema due to kidney function impairment, sepsis and organ failures. Further studies are warranted to comprehensively grasp the incidence of increased thickness and its potential implications for patient outcomes.

### Early extubation and outcomes association with lower diaphragm atrophy

Our study suggests that patients who underwent early extubation while assisted by VA-ECMO may have experienced protection against diaphragm atrophy. However, it is crucial to note that our study establishes an association rather than causality. Nevertheless, our results aligh with literature, where the removal of respiratory assistance has been associated with lower atrophy [20]. The recent proposition that extracorporeal life support could mitigate disuse atrophy was also formulated recently [22]. Although this finding would need confirmations using a gold standard procedure such as magnetic stimulation of the phrenic nerves and muscle histology, the novelty of our findings lies in the potential diaphragm protection through early extubation of VA-ECMO assisted patients with a personalized intervention. Further, our finding that diaphragm atrophy was associated with a higher mortality at two months is consistent with previous study from Goligher et al. [22]. Although our study did not specifically address whether mitigating diaphragm atrophy mediated better outcomes, this hypothesis that preserving survivors from a respiratory myotrauma could positively impact outcomes remains a challenging question that requires further studies.

### Contributors of diaphragm thickness evolution during VA-ECMO

In our analysis, we found that factors such as mechanical ventilation, early administration of neuromuscular blocking agents, and vasoactive amines as associated with diaphragm atrophy, aligning with existing literature [13]. However, neuromuscular blocking agents monitoring was not included in our analysis, limiting conclusive insights into their influence. Remarkably, only a small number of our patients presented with sepsis, a known promoter of diaphragm atrophy and dysfunction [30]. Interestingly, the Insulin daily doses were correlated with T_EE_ evolution, consistent with studies focusing on preserving muscle function through tight glycaemic control [31]. Although not confirmed in the MLM, this finding underscores the role of metabolic control.

Our findings suggest that diaphragm contractile activity, as measured by the thickening fraction, may not be directly correlated with the changes in diaphragm thickness during VA-ECMO treatment. We employed three statistical models, including univariable analysis, repeated measures correlation, and a MLM known for its efficiency in handling missing values, repeated measures, and factor variables simultaneously [28]. Also, the MLM permitted an analysis of T_EE_ with sequential variation of expositions during the study period. However, disregarding the contractile activity per se as a mechanism of myotrauma in the context of VA-ECMO may oversimplify the situation, as the dTF represents a third of respiratory efforts and exhibits individual variations [32, 33]. Thus, decoupling diaphragm contractile activity from changes in diaphragm thickness with VA-ECMO sweep gas flow may be a worthwhile pursuit to mitigate the impact of load-induced injuries in line with studies using neuromuscular blocking agents [19, 34].

The association of sweep gas flow with the T_EE_ evolution emphasizes the importance of its careful titration [18]. In our model, we also identified that the acidosis balance was involved in the T_EE_ evolution. Both factors are recognized contributors to homeostasis, with acidosis being a major contributor to respiratory drive [35], while sweep gas flow contributes to decarboxylation in extracorporeal life support [36].

### Diaphragm contractile activity in cardiogenic shock

#### Prevalence of low diaphragm contractile activity

In our study, the majority of patients demonstrated diaphragm contractile activity below 20% dTF until day 7 [8, 37]. These findings align with prior animal studies that indicated an initial reduction in contractility due to cardiogenic shock [17]. Specifically, over two-thirds of the patients exhibited a dTF < 20% during their first week of assistance, closely resembling recent findings in VV-ECMO patients. These findings were established using the gold standard for diaphragm function exploration, involving the change of endotracheal tube pressure induced by bilateral phrenic nerve stimulation during airway occlusion. This method was applied in highly sedated COVID-19 patients [38]. In the same paper, the contractility measured using the diaphragm thickening fraction method remained stable throughout the study period, unlike what was observed in VA-ECMO survivors.

#### Contributors of low diaphragm contractile activity in cardiogenic shock

This study did not reveal a clear association between low diaphragm contractile activity and potential risk factors. It is crucial to note that the effort condition varied throughout the study and the individual response to central drive output. However, along recent findings from our team [18], the sweep gas flow was associated with the thickening fraction evolution in the present study using MLM. In our previous paper, the systematic decrease in sweep gas flow was correlated with the diaphragm thickening fraction during a VA-ECMO weaning assessment [18]. Our results confirms the importance of careful sweep gas flow titration due to its interaction with diaphragm contractility.

#### Diaphragm contractile activity and outcomes

In the present study, no association between the contractile activity and patient outcomes was identified. Several factors could contribute to this finding. First, this study was not designed to evaluated the prognostic value of dTF on patient outcomes, lacking standardize weaning procedures, for instance. Secondly, existing literature does not consistently support the use of dTF as prognostic index for mechanical ventilation weaning either in high-risk ICU patient, those in cardiogenic shock, or undergoing cardiac surgery [14, 39, 40]. Thirdly, this longitudinal study involved critically ill patients with frequent multiple organ failure, leading to multifaceted treatment procedures and ever-evolving titration of the assistance. Consequently, respiratory efforts were constantly subject to numerous interfering factors, which was not the case of the diaphragm thickness.

### Diaphragm protection with VA-ECMO

This study provides evidence that low diaphragm contractility was observed in patient without diaphragm atrophy during VA-ECMO. The mechanism of diaphragm protection with low contractility in VA-ECMO patient are unclear. The preserved T_EE_ at low dTF might be controversial in light of recent findings advocating for maintaining contraction through stimulation devices [41]. Furthermore, a landmark study from Goligher and al found that preserved thickness was more frequent when patients’ thickening fraction varied in a range between 15 and 30% during mechanical ventilation [20]. Several factors can help reconcile this controversy. Firstly, the study populations differed, with a minority being septic patients known to promote diaphragm atrophy [30]. Secondly, VA-ECMO provided preserved hemodynamic and mitigated metabolic disorders. Thirdly, it is important to note that VA-ECMO bypasses the respiratory central command. Fourthly, patients were extubated early.

Revisiting the studies conducted by Aubier et al. in a cardiogenic shock model induced in dogs [17, 42, 43], they found that the initial increase in diaphragm effort was correlated with a decrease in oxygen delivery. The reduced cardiac output was associated with a decline in respiratory muscle blood flow, promoting anaerobic metabolic pathways, a situation alleviated by mechanical ventilation and muscle paralysis, both described as a promoter of diaphragm dysfunction induced by mechanical ventilation in later studies [11].

In summary, when shock is controlled with VA-ECMO, respiratory muscle blood flow might be sustained, resulting in minimal demand. Simultaneously, the downregulation from the central command might be intensified by the extracorporeal by-pass. In these circumstances, VA-ECMO may favour low contractile activity, early extubation and the preservation of diaphragm thickness changes.

### Strength and limitations

This study has notable strengths, including the use of a non-invasive technique for measuring diaphragm thickness and contractility, with data analyzed offline by a blinded operator. It is the first prospective study to assess diaphragm thickness changes in patients treated with extracorporeal life support.

However, there are several limitations. Firstly, the limited number of patients limited the inclusion of variables in the statistical analysis. Secondly, the study was observational and lacked a control group, making it challenging to determine the role of ventilation settings. Thirdly, the technique itself has limitations, especially regarding the reproducibility of thickness loss, particularly when using a 10% threshold. Fourthly, the study did not document the use of non-invasive ventilation or high-flow nasal cannula, considering all patients as extubated due to the small sample size. Fifthly, the evolution of diaphragm changes beyond one week of VA-ECMO initiation was not measured. Lastly, this was a single-center study, limiting its generalizability.

## Conclusion

In summary, fewer cases of progressive atrophy were described when early extubation was possible. The association between decrease in diaphragm thickness and lower mortality at two months needs further confirmation in larger studies. Variables related to cellular homeostasis and respiratory drive—i.e. acidosis and sweep gas flow—were linked to diaphragm structural modifications as assessed by ultrasound. The preserved diaphragm thickness in VA-ECMO survivors highlights the importance of implementing diaphragm-protective strategies, emphasizing the role of tight pH control through sweep gas flow titration for patients with cardiogenic shock.

### Supplementary Information

**Additional file 1:Table 1.** Included cardiopathy, type of surgery procedures and septic status in the studied population.
**Table 2.** Patients characteristics according to their liberation from mechanical ventilation status at day 3.
** Table 3.** Patients characteristics according to their diaphragm evolution status at the end of patient follow-up.
**Table 4.** Diaphragm thickness at end expiration evolution in cardiogenic shock treated with VA-ECMO: mixed-linear model and sensitivity analysis.
**Table 5.** Diaphragm thickening fraction (dTF %) evolution in patient extubated before day 4 or not.
**Table 6.** Patients characteristics according to the diaphragm contractile activity.
**Table 7.** Diaphragm thickening fraction evolution in cardiogenic shock treated with VA-ECMO: calculation of a mixed-effect linear model (MLM) of diaphragm thickness over time, with subjects treated as random effects.
**Table 8.** Patients outcome according to their liberation from mechanical ventilation status at day 3
**Table 9.** Outcome of the studied population according to the contractile activity status.
**Table 10.** Univariate analysis of death at 60 days. Logistic regression model of the risk of death at 60 days adjusted for age, body mass index, cardiac arrest, SAPS2 and the presence of diaphragm atrophy by day 7.
**Table 11.** Characteristics of patients deceased during the 60 days follow-up after the implantation of a VA-ECMO.
**Figure 1.** Study flowchart.
**Figure 2.** Graphic representations of the individual values of thickness evolution.
**Figure 3.** Graphic representations of repeated measure correlation.
**Figure 4.** pH values in VA-ECMO in intubated or extubated patients by day three.
**Figure 5.**Sweep gas flow values in VA-ECMO in intubated or extubated patients by day three.
**Figure 6.** PaCO2 (mmHg) values in intubated or extubated patients by day three.
**Figure 7.**VA-ECMO flow values in intubated or extubated patients by day three.
**Figure 8.**pH evolution according the sweep gas flow according the three groups of thickness classification.
**Additional file 1:**
**Figure 9.**visual abstract

## Data Availability

The datasets used and/or analysed during the current study are available from the corresponding author on reasonable request.

## Abbreviations

95% CI: 95% Confidence Interval
dTF: Diaphragm thickening fraction
95% CI: 95% Confidence Interval
FiO_2_: Oxygen inspired fraction
ICU: Intensive care unit
KDIGO: Kidney Disease Improving Global Outcomes
MLM: Mixed-effect linear model
LVEF: Left ventricle ejection fraction
NIV: Non-invasive ventilation
OR: Odds ratio
PEEP: Positive end expiratory pressure
RASS: Richmond agitation sedation scale
RMCr: Repeated measure correlation
RMCr: Repeated measure correlation
SAPS II: Severe acute physiology score II
SOFA: Severity of organ failure assessment
T_EE_: Diaphragm thickness at end expiration
T_Ei_: Diaphragm thickness at end inspiration
VA-ECMO: Veno-arterial extracorporeal membrane oxygenation
VV-ECMO: Veno-Venous extracorporeal membrane oxygenation
