# Supplementary material for: Diaphragm thickness modifications and associated factors during VA-ECMO for a cardiogenic shock: a cohort study
Source: Ann Intensive Care. 2024 Mar 8;14:38. doi: 10.1186/s13613-024-01264-8 (PMC10923772; doi:10.1186/s13613-024-01264-8)

Diaphragm thickness modifications and associated factors during VA-ECMO for a cardiogenic shock: a cohort study

Pierre-Henri Moury [1,2], MD PhD ; Alexandre Béhouche[1], MD ; Sébastien Bailly[2], PhD ; Zoé Durand[1], MD; Géraldine Dessertaine[1], MD ; Angelina Pollet[1], Msc; Samir Jaber[3], MD PhD ; Samuel Verges[2], PhD ; Pierre Albaladejo[1], MD PhD

Additional materials section

Table of contents

METHODS 2
Diaphragm assessment and study procedure 2
Mechanical ventilation, ECMO care, and patient outcome 2
Pharmacological drug use 4
Statistical analysis 4
Primary analysis and variable choice in the mixed-effect linear model (MLM) 4
Sensitivity analaysis 5
RESULTS 6
Patients' characteristics 6
Early extubation 7
Low diaphragm contractile activity 7
Patient outcome 8
Visual abstract 8
REFERENCES 9
Table S1: Included cardiopathy, type of surgery procedures, and septic status in the studied population 10
Table S2: Patients' characteristics according to their liberation from mechanical ventilation status at day 3. 11
Table S3: Patient characteristics 13
Table S4: Diaphragm thickness at end-expiration evolution in cardiogenic shock treated with VA-ECMO: mixed-linear model and sensitivity analysis 14
Table S5: Diaphragm thickening fraction (dTF %) evolution in patient extubated before day 4 or not. 15
Table S6: Patient characteristics according to the diaphragm contractile activity. 16
Table S7: Diaphragm thickening fraction evolution in cardiogenic shock treated with VA-ECMO: mixed-linear model 17
Table S8: Patients outcome according to their liberation from mechanical ventilation status at day 3 18
Table S9: Outcome of the studied population according to the contractile activity status. 19
Table S10: Univariate analysis of death at 60 days. 20
Table S11: Characteristics of patients deceased during the 60-day follow-up after the implantation of a VA-ECMO 21
Figure S1: Study flowchart. 23
Figure S2: Graphic representations of the individual values of thickness evolution 24
Figure S3: Graphic representations of repeated measure correlation 25
Figure S4: pH values in VA-ECMO in intubated or extubated patients by day three 27
Figure S5: Sweep gas flow values in VA-ECMO in intubated or extubated patients by day three 28
Figure S6: $PaCO_2$ (mmHg) values in intubated or extubated patients by day three 29
Figure S7: VA-ECMO flow values in intubated or extubated patients by day three 30
Figure S8: pH evolution according to the sweep gas flow according to the three groups of thickness classification. 31
Figure S9: Visual abstract 32

METHODS

Diaphragm assessment and study procedure

Ultrasound were conducted by a trained physician with validated experience in diaphragm assessment.[1–3]. Ultrasound measurements were conducted independently through computer-driven software on images extracted from the database (DICOM viewer 3.0, Philips, Netherlands). Ultrasound measurements were recorded, measured, and averaged over at least three respiratory cycles. Diaphragm thickness measurements were taken in a 20-30° upright position in the zone of apposition of the diaphragm to the rib cage and the liver, specifically between the 8th-10th intercostal spaces using a 7.5-12 MHz probe [4,5]. Thickness measurements were conducted both at the end of inspiration (T_{EI}, mm) and expiration (T_{EE}, mm). The diaphragm thickening fraction (dTF) was calculated as $(T_{EI}-T_{EE})/T_{EE}$ and expressed as a percentage. If a trained investigator was absent, the ultrasound measurement was not performed.

Mechanical ventilation, ECMO care, and patient outcome

Mechanical ventilation was initiated in control modes to ensure a tidal volume below the target of 6ml/kg of predicted ideal body weight and below the 14 cmH2O driving pressure threshold. The VA-ECMO blender was systematically used to set the sweep gas flow and VA-ECMO membrane Oxygen fraction: the Fraction of delivered oxygen (FDO2). The VA-ECMO sweep gas flow was set to provide an appropriate pH and $PaCO_2$ balance according to the tidal volume target and the driving pressure target in intubated and extubated patients. A lower threshold of 1.5 L/min for the sweep gas flow was observed to prevent hypoxemia resulting from the veno-arterial shunt. Pressure support ventilation was adjusted to achieve the same levels of protective mechanical ventilation. Positive end-expiratory pressure (PEEP) and an oxygen-inspired fraction (FiO_2) were adapted based on hypoxemia levels and tolerance. The Fraction of

delivered oxygen (FDO2) was set according to the FiO_2 levels. Weaning from mechanical ventilation in ECMO patients was assessed daily to facilitate early extubation, which was considered a standard of care in the unit.

Patients were extubated if they succeeded in a spontaneous breathing trial with appropriate blood gases, and favorable neurological status, devoid of discomfort, signs of major neurological injuries, or coma (Glasgow coma scale ≤9). High-flow nasal oxygenation (HFNO) or non-invasive ventilation (NIV) were utilized as needed. Reintubation occurred in cases of respiratory, neurological, or worsening cardiovascular failure [6,7].

Patients' assistance and outcomes were categorized into the following:

- VA-ECMO and mechanical ventilation in controlled modes
- VA-ECMO and mechanical ventilation in pressure support ventilation with spontaneous breathing modes
- VA-ECMO and extubated patients
- Patients weaned from VA-ECMO and assisted by mechanical ventilation in controlled modes
- Patients weaned from VA-ECMO and assisted by mechanical ventilation in pressure support ventilation with spontaneous breathing modes
- Patients fully weaned
- Patients deceased

Ventilator status was recorded both as a categorical variable with the previously mentioned categories and as a binary variable indicating the intubation status. The number of days ventilated and ventilator-free days at 60 days were recorded. Weaning from ECMO was decided if there was no onset of new respiratory, neurological, or cardiovascular failure that was clinically relevant. All patients were on peripheral VA-ECMO.

Early extubation was defined as liberation from mechanical ventilation before day 4. Factors associated with early extubation are described in the additional materials.

Pharmacological drug use

Neuromuscular blocking agents (NMBA) were used in case of refractory hypoxemia, major asynchronies, and during induced hypothermia for neuroprotection purposes after cardiac arrest. Norepinephrine was used in case of hypotension defined by a mean arterial pressure below 65mmHg despite VA-CMO optimization.

Statistical analysis

Sample size calculation

Sample size calculation was performed based on preliminary clinical results. A maximum of these five variables was included in the linear mixed model accounting for six participants for each variable. Consequently, thirty patients were needed. Also, we accounted for a maximum loss of 50% of the observations of the primary endpoint (diaphragm thickness) due to the death of patients before the end of the first week and the impossibility of including patients on weekends. The expected number of observations was 105 ultrasound measurements of the diaphragm thickness.

Primary analysis and variable choice in the mixed-effect linear model (MLM)

Variables were chosen according to their physiological importance and if they had a P-value of less than 0.1 either from univariable analysis or repeated-measure correlation.

The analysis of a mixed-effect linear model (MLM) of diaphragm thickness over time was used, with subjects treated as random effects.[8] Logit transformations were used to normalize values included in the MLM to improve the stability of the model.[8] The final model was chosen according to the parsimony principle and comparison between each Akaike information

criterion (AIC). In a secondary analysis, we also used an MLM to characterize influential factors of dTF.

We used the simulation DHARMa package from R software to test if the models were overdispersed using a two-sided method with 0.05 as the p-value threshold, Florian Hartig (2022).

Sensitivity analaysis

We conducted an unplanned sensitivity analysis by comparing two subsets of the original cohort. Two specific analyses were performed:

1. **Comparison of patients with atrophy to those without atrophy regardless of thickness increase:**
    - This analysis involved comparing the group of patients with atrophy to those without atrophy, irrespective of the existence of an increase in thickness.
2. **Two subsets population analyses:**
    - **Subset 1:** Exclusion of patients without a history of oro-tracheal intubation.
    - **Subset 2:** Removal of patients who experienced less than 24 hours of oro-tracheal intubation and were never assessed while intubated. This subset included patients without a history of measurement during mechanical ventilation (N=6).

These sensitivity analyses aimed to explore the robustness of the findings and assess whether the results were consistent across different patient subsets. The analyses were performed using the final Mixed Linear Model (MLM) on the specified subsets of the original cohort.

Complementary analysis

Marginal R^2 and conditional R^2 were calculated.

We completed the analysis in the additional material by displaying the characteristics of patients according to their extubation status on day 3 (table S2), the VA-ECMO parameters (figure S3), and the blood gases analysis.

RESULTS

Patients' characteristics

The patients' type of surgical procedure, whether they had a myocardial arrest or not, and the presence of sepsis are displayed in Table S1. The study flowchart of this study marked by the COVID-19 pandemic impact on the inclusion is described in figure S1. Patients' characteristics according to their liberation from mechanical ventilation status on day 3 are displayed in Table S2. Table S3 describes the complementary characteristics according to the three group categories (increase, stable, and atrophy) and two group categories (atrophy and non-atrophy). The thickness of the diaphragm at end-expiration (T_{EE}) was measured at 2.8 mm [2.5; 3.2] in the study population on the day of inclusion and was measured at 2.8 mm [2.5; 3] at the end of follow-up on day 7. The minimum T_{EE} measured was 1.8mm meaning that no patients were below the 1.5 mm threshold recognized for pre-existing neuromuscular involvement in a diaphragm atrophy [9]. Individual values are described in the figure S2.

The repeated measure correlation (RMCc) concerning pH, Sweep gas flow, and Insulin are described in figure S3. The repeated measure correlation between and the fluid balance was not significant (RMCc = 0.07, 95% CI: [-0.13; 0.27], p-value = 0.5).

The diaphragm thickness at the end-expiration evolution model in cardiogenic shock treated with VA-ECMO and sensitivity analysis using the mixed-effect linear model are shown in Table S4. The marginal R^2 was 0.173 and the conditional R^2 was 0.714.

Early extubation

None of the patients liberated from mechanical ventilation by day 3 or without intubation after inclusion (2 patients) experienced diaphragm atrophy at the end of the first week. Characteristics' of the patients' according to their early extubation status on day 3 are described in Table S2.

Over the three first days after inclusion and a VA-ECMO treatment for a cardiogenic shock, patients who were extubated displayed significantly different blood gases and VA-ECMO parameters.

Alkalosis was more frequent in extubated patient (7.48 [7.46, 7.49] versus 7.38 [7.36, 7.43)] in intubated patient; p-value = 0.006), see figure S4, at a significantly lower sweep gas flow (4 l/min [4, 5] intubated patient versus 3 l/min [2,3] in extubated patient, p-value=0.002), see figure S5, while the $PaCO_2$ was also lower in extubated patient (42 mmHg [39, 44] in intubated versus 38 mmHg [38, 39] in extubated; p-value = 0.024), see figure S6. The VA-ECMO output is a major contributor to PaO_2 [10] and showed no significant difference at 4 l/min [3, 5] in intubated patients versus 3 l/min [3, 4] (p-value=0.2), see Figure S7. None of the patients extubated by day 3 deceased at 60 days (p-value <0.001).

Low diaphragm contractile activity

The low diaphragm contractile activity was defined by a dTF < 20%. Patients were divided into two populations if they reached the 20% threshold at least one time during the study period. Overall, 69% of the studied population (20 patients) had a dTF throughout the study period. The characteristics of the patients according to the low diaphragmatic contractile activity threshold are shown in Table S6. Patients who presented a low diaphragm contractile activity did not significantly encounter adverse outcomes. The diaphragm thickening fraction evolution model in cardiogenic shock treated with VA-ECMO is described in Table S7. The inclusion of

daily fluid balance in the MLM revealed an association between dTF and sweep gas flow (Beta = -2.8; 95% CI [-5.2, -0.5], p-value =0.017).

Patient outcome

The outcome is described in Table S8 according to the liberation status at day 3 and in Table S9 according to the dTF threshold of 20. The logistic regression model of the risk of death at 60 days is presented in Table 10. The model was adjusted for age, body mass index, cardiac arrest, SAPS2, extubation at day 3 status, and the presence of diaphragm atrophy by day 7. The description of the patient extubated by the end of the third day is described in Table S2. We described the patients who deceased during the two-month follow-up period in Table S11.

Visual abstract

A visual abstract is provided in Figure S9.

REFERENCES

1. Moury P-H, Cuisinier A, Durand M, Bosson J-L, Chavanon O, Payen J-F, et al. Diaphragm thickening in cardiac surgery: a perioperative prospective ultrasound study. Ann Intensive Care. 2019;9:50.

2. Moury PH. Diaphragm Thickening During Venoarterial Extracorporeal Membrane Oxygenation Weaning: An Observational Prospective Study. Journal of Cardiothoracic and Vascular Anesthesia. 2021;8.

3. Haaksma ME, Smit JM, Boussuges A, Demoule A, Dres M, Ferrari G, et al. EXpert consensus On Diaphragm UltraSonography in the critically ill (EXODUS): a Delphi consensus statement on the measurement of diaphragm ultrasound-derived parameters in a critical care setting. Crit Care. 2022;26:99.

4. Gottesman E, McCool FD. Ultrasound evaluation of the paralyzed diaphragm. American journal of respiratory and critical care medicine. 1997;155:1570–4.

5. Matamis D, Soilemezi E, Tsagourias M, Akoumianaki E, Dimassi S, Boroli F, et al. Sonographic evaluation of the diaphragm in critically ill patients. Technique and clinical applications. Intensive Care Med. 2013;39:801–10.

6. Thille AW, Richard J-CM, Brochard L. The Decision to Extubate in the Intensive Care Unit. American Journal of Respiratory and Critical Care Medicine. 2013;187:1294–302.

7. Boles J-M, Bion J, Connors A, Herridge M, Marsh B, Melot C, et al. Weaning from mechanical ventilation. European Respiratory Journal. 2007;29:1033–56.

8. Harrison XA, Donaldson L, Correa-Cano ME, Evans J, Fisher DN, Goodwin CED, et al. A brief introduction to mixed effects modelling and multi-model inference in ecology. PeerJ. 2018;6:e4794.

9. McCool FD, Tzelepis GE. Dysfunction of the Diaphragm. New England Journal of Medicine. 2012;366:932–42.

10. Schmidt M, Tachon G, Devilliers C, Muller G, Hekimian G, Bréchot N, et al. Blood oxygenation and decarboxylation determinants during venovenous ECMO for respiratory failure in adults. Intensive Care Med. 2013;39:838–46.

Table S1: Included cardiopathy, type of surgery procedures, and septic status in the studied population

Patient	Cardiopathy	Myocardial arrest	Type of surgery	Sepsis
001	Ischemic	No	CABG	No
002	Cardiogenic shock	No	NA	yes
003	Ischemic	Yes	NA	No
004	valvular	No	Aortic valve	No
005	Ischemic + valvular	No	Aortic valve	No
006	Valvular and aortic	No	Bentall procedure	No
007	Ischemic	Yes	NA	No
008	cardiomyopathy	No	NA	No
009	NA	Yes	NA	No
010	Ischemic	No	CABG	No
011	Ischemic	No	CABG	No
012	Ischemic + cardiomyopathy	Yes	Heart transplantation	No
013	valvular	No	Aortic valve	No
014	cardiomyopathy	No	NA	No
015	Ischemic	Yes	NA	No
016	Ischemic	Yes	NA	No
017	valvular	No	Aortic valve/endocarditis	yes
018	Ischemic	No	CABG	No
019	Ischemic	Yes	NA	No
020	Ischemic	Yes	NA	No
021	cardiomyopathy	No	NA	No
022	Ischemic	No	NA	No
023	Ischemic + cardiomyopathy	No	Mitral valve + CABG	No
024	NA	Yes	NA	No
025	Cardiogenic shock	Yes	NA	No
027	Ischemic	Yes	NA	No
028	Respiratory failure/ pulmonary embolism	Yes	NA	No
029	Ischemic/ventricular septal defect	No	NA	No
030	valvular	No	Aortic valve	No
CABG: Cardionary artery bypass graft				

Table S2: Patients' characteristics according to their liberation from mechanical ventilation status at day 3.

Variable	N	Patients extubated or not > day 3, N = 16[1]	Patients extubated and not ventilated ≤ day 3, N = 13[1]	p-value[2]
**Age (years)**	29	54 [48, 69]	64 [48, 66]	0.8
**Sexe**	29	14 [88%]	11 [85%]	>0.9
**Weight (kg)**	29	85 [74, 90]	70 [63, 91]	0.3
**Heigth (m)**	29	1.71 [1.7, 1.8]	1.75 [1.7, 1.8]	0.7
**Body Mass Index**	29	27 [25, 29]	24 [21, 29]	0.10
**SOFA score**	29	8 [8, 11]	9 [4, 10]	0.5
**Severe Acute Physiology Score II**	29	50 [46, 60]	41 [34, 43]	<0.001
**Cardiac arrest**	29	7 (44%)	5 (38%)	0.8
**LVEF at the time of VA ECMO implantation**	29	10 (4, 16)	10 (5, 15)	0.8
**Cardiac surgery**	29	7 (44%)	5 (38%)	0.8
**Chronic obstructive Pulmonary Disease**	29	1 (6.2%)	1 (7.7%)	>0.9
**Cancer**	29	0 (0%)	2 (15%)	0.2
**Chronic kidney insufficiency**	29	1 (6.2%)	0 (0%)	>0.9
**Sepsis**	29	1 (6.2%)	1 (7.7%)	>0.9
Mechanical ventilation parameters (All following parameters were averaged over the three first days)				
**Positive end-expiratory pressure (cmH2O)**	23	9 [7, 10]	8 [8, 9]	>0.9
**Respiratory rate (cycle/min)**	28	14 [13, 16]	19 [14, 20]	0.017

Variable	N	Patients extubated or not > day 3, N = 16[1]	Patients extubated and not ventilated ≤ day 3, N = 13[1]	p-value[2]
**TV (ml)**	24	341 [295, 404]	373 [344, 419]	0.2
**Pharmacological parameters** ***(All following parameters were averaged over the three first days)***				
**Norepinephrine (mg/day)**	29	93 [22, 100]	2 [0, 12]	<0.001
**Dobutamine (mg/day)**	29	176 [107, 250]	98 [9, 161]	0.11
**Insulin (Units/days)**	29	25 [11, 38]	7 [0, 40]	0.2
**Propofol (mg/day)**	29	1,973 [1,455, 3,273]	192 [0, 1,439]	0.002
**Neuromuscular blocking agent**	29	12 (75%)	3 (23%)	0.005
**Corticosteroids**	29	2 (12%)	1 (7.7%)	>0.9

[1]Median [IQR]; n (%)

[2]Wilcoxon rank sum test; Fisher's exact test; Pearson's Chi-squared test; Wilcoxon rank sum exact test

SOFA: Sepsis-related Organ Failure Assessment; *VA-ECMO:* Veno-arterial extracorporeal membrane oxygenation; *LVEF:* Left Ventricle Ejection Fraction; *TV*: Tidal volume.

Table S3: Patient characteristics

Results are reported for patients according to their diaphragm evolution status at the end of patient follow-up. We divided the study population into three categories whether the diaphragm thickness presented a ≥10% decrease, increase, or stability from baseline to the last assessment and according to the presence or absence of diaphragm atrophy.

	**Patient characteristics according to three groups of diaphragm thickness evolution (: stable, atrophy, increase) using an ultrasound method**				**Patient characteristics according to the absence or the presence of diaphragm atrophy using an ultrasound method**		
**Variable**	**Increase**, N = 4[1]	**Stable**, N = 18[1]	**Atrophy**, N = 7[1]	**p-value**[2]	**Non-atrophy**, N = 22[1]	**Atrophy**, N = 7[1]	**p-value**[3]
Mechanical ventilation parameters (All following parameters were averaged over the three first days)							
**Positive end-expiratory pressure (cmH2O)**	8 [8, 8]	9 [7, 10]	9 [7, 11]	>0.9	8 [8, 10]	9 [7, 11]	0.9
**Plateau pressure (cmH2O)**	16 [16, 16]	16 [12, 19]	13 [10, 18]	0.6	16 [13, 19]	13 [10, 18]	0.4
**Driving pressure (cmH2O)**	8 [8, 8]	6 [5, 9]	4 [3, 6]	0.4	6 [5, 9]	4 [3, 6]	0.2
**Respiratory rate (cycle/min)**	16 [12, 20]	15 [13, 18]	14 [14, 17]	>0.9	15 [13, 19]	14 [14, 17]	>0.9
**TV (ml)**	343 [335, 359]	345 [309, 395]	407 [327, 451]	0.6	345 [321, 375]	407 [327, 451]	0.4
Pharmacological parameters (All following parameters were averaged over the three first days)							
**Norepinephrine (mg/day)**	7 [1, 20]	14 [0, 59]	98 [58, 118]	0.028	12 [0, 41]	98 [58, 118]	0.009
**Dobutamine (mg/day)**	241 [152, 279]	215 [70, 274]	197 [61, 350]	>0.9	215 [70, 278]	197 [61, 350]	>0.9
**Insulin (Units/day)**	37 [25, 43]	13 [1, 35]	26 [16, 33]	0.3	19 [2, 39]	26 [16, 33]	0.5
**Propofol (mg/day)**	1,587 [1,141, 1,667]	1,368 [56, 2,931]	1,980 [1,496, 2,920]	0.3	1,390 [56, 1,902]	1,980 [1,496, 2,920]	0.13
**NMBA**	0 (0%)	9 (50%)	6 (86%)	0.021	9 (41%)	6 (86%)	0.080
**Corticosteroids**	1 (25%)	1 (5.6%)	1 (14%)	0.3	2 (9.1%)	1 (14%)	>0.9
Blood gas parameters (All following parameters were averaged over the three first days)							
**pH**	7.47 [7.43, 7.48]	7.44 [7.39, 7.49]	7.38 [7.36, 7.45]	0.4	7.45 [7.39, 7.49]	7.38 [7.36, 7.45]	0.2
**PaO2 (mmHg)**	70 [67, 75]	82 [76, 93]	93 [67, 100]	0.2	82 [74, 91]	93 [67, 100]	0.8
**PaCO2(mmHg)**	41 [39, 44]	39 [37, 41]	40 [39, 42]	0.2	39 [38, 42]	40 [39, 42]	0.2

[1]Median (IQR); n (%); [2]Kruskal-Wallis rank sum test; Fisher's exact test; [3]Wilcoxon rank sum test; Fisher's exact test; Wilcoxon rank sum exact test

BMI: Body mass index; *SAPS2:* Simplified Acute Physiology Score 2*; VA-ECMO:* Veno-arterial extracorporeal membrane oxygenation; *LVEF:* Left Ventricle Ejection Fraction; *TV*: Tidal volume. NMBA: neuromuscular blocking agents; ;[2]Kruskal-Wallis rank sum test; Fisher's exact test

Table S4: Diaphragm thickness at end-expiration evolution in cardiogenic shock treated with VA-ECMO: mixed-linear model and sensitivity analysis

Primary analysis used a mixed-effect linear model (MLM) of diaphragm thickness over time, with subjects treated as random effects.[8] The final model was applied to two subsets of the population. First, patients without any history of oro-tracheal intubation (N=2) were excluded. Second, patients without a history of measurement during mechanical ventilation were included (N=6). Simulation DHARMa package from R software was used to test the model's overdispersion, Florian Hartig (2022).

	The final model in the complete population, N=29			Exclusion of patients without a history of intubation, N=27			Exclusion of patients without a history of intubation intubated < 24 hours		
**Characteristic**	**Beta**	**95% CI**[1]	**p-value**	**Beta**	**95% CI**[1]	**p-value**	**Beta**	**95% CI**[1]	**p-value**
pH	-2.0	-2.9, -1.1	<0.001	-2.1	-3.0, -1.1	<0.001	-2.5	-3.5, -1.5	<0.001
Diaphragm thickening fraction (%)	0.00	0.00, 0.01	0.8	0.00	-0.01, 0.01	>0.9	0.00	-0.01, 0.00	0.6
Sweep gas flow (L/min)	-3.0	-4.8, -1.2	0.001	-3.1	-4.8, -1.3	<0.001	-3.6	-5.5, -1.7	<0.001
Time (days)	0.00	-0.03, 0.02	0.8	-0.01	-0.04, 0.02	0.4	-0.01	-0.03, 0.02	0.7
ECMO days (factor)	-0.13	-0.28, 0.02	0.093	-0.18	-0.36, 0.00	0.054	-0.21	-0.40, -0.03	0.024
Interactions									
pH * Sweep gas flow	0.41	0.17, 0.64	<0.001	0.41	0.18, 0.65	<0.001	0.49	0.24, 0.75	<0.001
Diaphragm thickening fraction * Time	0.00	0.00, 0.00	0.5	0.00	0.00, 0.00	0.3	0.00	0.00, 0.00	0.14
**Overdispersion was tested with the p-value threshold of 0.05**		0.824			0.888			0.72	

[1]CI = Confidence Interval; *VA-ECMO:* Veno-arterial extracorporeal membrane oxygenation

Table S5: Diaphragm thickening fraction (dTF %) evolution in patient extubated before day 4 or not.

**Diaphragm thickening fraction (%)**	**N**	**Patients extubated on day 4 and after**, n = 16[1]	**Patients extubated before day 4**, n = 13[1]	**p-value**[2]
**Day 1, %**	28	3 [0, 6]	11 [6, 15]	0.078
**Day 2, %**	25	4 [0, 8]	9 [4, 11]	0.051
**Day 3, %**	25	4 [0, 10]	12 [12, 15]	0.007
**Day 4, %**	22	4 [0, 9]	11 [7, 19]	0.061
**Day 5, %**	17	11 [7, 11]	10 [7, 15]	0.9
**Day 6, %**	10	17 [11, 27]	15 [12, 19]	>0.9
**Day 7, %**	6	21 [20, 23]	20 [15, 36]	>0.9

[1]Median (IQR)

[2]Wilcoxon rank sum test; Wilcoxon rank sum exact test

Table S6: Patient characteristics according to the diaphragm contractile activity.

We defined by a diaphragm thickening fraction below 20% a low contractile activity.

Variable	**Diaphragm thickening fraction ≤ 20%**, N = 20[1]	**Diaphragm thickening fraction > 20%**, N = 9[1]	p-value[2]
**Age (years)**	56 [48, 66]	59 [51, 68]	0.6
**Sexe (male)**	19 [95%]	6 [67%]	0.076
**Weight (kg)**	85 [73, 93]	70 [63, 84]	0.14
**Height (m)**	1.74 [1.70, 1.80]	1.75 [1.69, 1.80]	0.6
**Body Mass Index (kg/m²)**	26 [25, 29]	24 [21, 27]	0.2
**SOFA score**	9 [7, 10]	9 [8, 13]	0.5
**Severe Acute Physiology Score II**	48 [42, 52]	42 [34, 43]	0.081
**Cardiac arrest**	7 [35%]	5 [56%]	0.4
**LVEF at the time of VA ECMO implantation**	10 [5, 23]	10 [5, 10]	0.5
**Cardiac surgery**	8 (40%)	4 (44%)	>0.9
**Chronic obstructive pulmonary disease**	1 (5%)	1 (11%)	0.5
**Chronic kidney insufficiency**	1 (5%)	0 (0%)	>0.9
**Sepsis**	1 (5%)	1 (11%)	0.5

[1]Median (IQR); n (%)[2]Wilcoxon rank sum test; Fisher's exact test; Wilcoxon rank sum exact test

LVEF: Left Ventricle Ejection Fraction; *VA-ECMO:* Veno-Arterial Extracorporeal Membrane Oxygenation

Table S7: Diaphragm thickening fraction evolution in cardiogenic shock treated with VA-ECMO: mixed-linear model

The primary analysis was the calculation of a mixed-effect linear model (MLM) of diaphragm thickness over time, with subjects treated as random effects.[8]

**Characteristic**	**Beta**	**95% CI**[1]	**p-value**
VA-ECMO days (as a binary factorial variable)	3.1	-8.2, 14	0.6
Mechanical ventilation days (as a binary factorial variable)	1.8	-4.8, 8.3	0.6
Sweep gas flow	-2.8	-5.2, -0.51	0.017
Daily fluid balance (ml)	0.00	-0.01, 0.00	0.4
**Interaction**			
Sweep gas flow. * Daily fluid balance	0.00	0.00, 0.00	0.8

[1]CI = Confidence Interval

Table S8: Patients outcome according to their liberation from mechanical ventilation status at day 3

Variable	Patient extubated or not > day 3, N = 16[1]	Patient extubated ≤ day 3, N = 13[1]	p-value[2]
**VA-ECMO days**	5.5 [3, 8)	6.0 [4.0, 10)	0.3
**Days with mechanical ventilation**	8.0 [5.8, 12)	1.0 [1, 6)	0.006
**Ventilator-free days at day 60**	9.0 [0, 48.2)	59 [54, 59)	<0.001
**Deceased at day 60**	10 (62%)	0 (0%)	<0.001
**Deceased on day 7**	5 (31%)	0 (0%)	0.048
**Group of diaphragm evolution**			0.010
Augmented (>10% increase in thickness)	1 (6.2%)	3 (23%)	
Atrophy (>10% decrease in thickness)	7 (44%)	0 (0%)	
Stable	8 (50%)	10 (77%)	
**Patients without atrophy**	9 (56%)	13 (100%)	0.008

[1]n (%); Median (IQR)

[2]Fisher's exact test; Pearson's Chi-squared test; Wilcoxon rank sum test

Table S9: Outcome of the studied population according to the contractile activity status.

We defined the low contractile activity by a diaphragm thickening fraction below 20%.

Variable	*N*	***Diaphragm thickening fraction ≤ 20%***, *N = 20*[1]	***Diaphragm thickening fraction > 20%***, *N = 9*[1]	*p-value*[2]
VA-ECMO duration (days)	*29*	*6 [1, 9]*	*6 [5, 12]*	*0.5*
Days with mechanical ventilation	*29*	*50 [0, 59]*	*54 [24, 55]*	*0.4*
Ventilator-free days at day 60	*29*	*8 (40%)*	*2 (22%)*	*0.7*
Deceased at day 60	*29*	*8 (40%)*	*2 (22%)*	*0.4*

[1]*n (%); Median (IQR)*

Table S10: Univariate analysis of death at 60 days.

Logistic regression model of the risk of death at 60 days adjusted for age, body mass index, cardiac arrest, SAPS2, and the presence of diaphragm atrophy by day 7.

Characteristics	OR[1]	95% CI[1]	p-value
**Age**	1.03	0.97, 1.10	0.33
**Body mass index**	1.09	0.97, 1.29	0.14
**Cardiac arrest**	0.48	0.08, 2.30	0.36
**SAPS 2**	1.18	1.06, 1.42	**<0.001**
**Patient with diaphragm atrophy by day 7**	8.50	1.39, 74.1	**0.020**
**The patient was extubated on Day 3**	0		**<0.001**

[1]OR = Odds Ratio, CI = Confidence Interval

SAPS2: Simplified Acute Physiology Score 2*; VA-ECMO:* Veno-arterial extracorporeal membrane oxygenation

Table S11: Characteristics of patients deceased during the 60-day follow-up after the implantation of a VA-ECMO.

Variable	**Survived two months after a VA-ECMO-assisted cardiogenic shock**, N = 19[1]	**Deceased two months after a VA-ECMO assisted cardiogenic shock**, N = 10[1]	p-value[2]
**Age (years)**	57 [48, 65]	62.0 [48.8, 73.8]	0.3
**Sexe**	16 [84%]	9 [90%]	>0.9
**Body Mass Index**	26 [23, 28]	27 [25, 30]	0.2
**SOFA**	8 [5, 10]	9 [8, 14]	0.072
**Severe Acute Physiology Score II**	42 [35, 46]	55 [47, 73]	0.004
**Cardiac arrest**	9 (47%)	3 (30%)	0.4
**LVEF at the time of VA ECMO implantation**	15 [7, 17]	7 [1, 10]	0.072
**Cardiac surgery**	7 (37%)	5 (50%)	0.7
**Sepsis**	1 (5.3%)	1 (10%)	>0.9
**Patient with diaphragm atrophy (day 7)**	2 (11%)	5 (50%)	0.030
**Mechanical ventilation on day 7**	1 (1, 4)	6 (5, 7)	0.001
**Patients extubated by day 4**	13 (68%)	0 (0%)	<0.001
**Dobutamine (mg/day)**	98 [5, 158]	231 [185, 266]	0.003
**Insulin (Units/days)**	17.1 [2.1, 40.6]	25.0 [14.2, 31.5]	0.7
**Propofol (mg/day)**	1,393 [5, 2,584]	1,519 [1,381, 2,474]	0.2
**Neuromuscular blocking agent**	7 [37%]	8 [80%]	0.050

Variable	Survived two months after a VA-ECMO-assisted cardiogenic shock, N = 19[1]	Deceased two months after a VA-ECMO assisted cardiogenic shock, N = 10[1]	p-value[2]
**pH**	7.47 [7.43, 7.49]	7.37 [7.36, 7.42]	0.043
**PaO2 (mmHg)**	82 [72.5, 92.5]	82.2 [73.7, 102.5]	0.5
**PaCO2(mmHg)**	39 [38, 39]	42 [40, 44]	0.017
**VA-ECMO Flow (l/min)**	3.0 [2.5, 4.0]	3.5 [3.1, 5]	0.2
**Sweep gas flow (l/min)**	3 [2.3, 3.8]	4.3 [3.7, 5.]	0.021

VA-ECMO: Veno-arterial extracorporeal membrane oxygenation; *LVEF:* Left Ventricle Ejection Fraction; *TV*: Tidal volume.

Figure S1: Study flowchart.

During the study period, due to the reorganization of the services during the COVID-19 pandemic, a trained ultrasound operator was possibly present during the first epoch of ten months, a second of three months, and a third of five months for a total of 18 months. Among the 61 patients implanted with an ECMO during this period, 10 were moribund at admission, 5 had a VV-ECMO, and six were admitted during the absence of the trained operator.

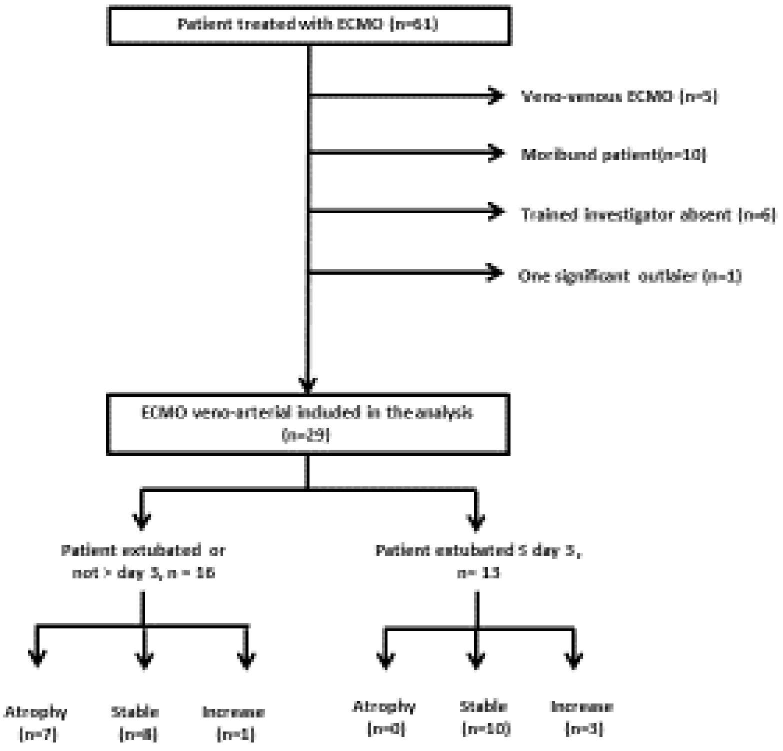

Figure S2: Graphic representations of the individual values of thickness evolution

The individual values are reported according to the three groups of diaphragm evolution (increase, stability, or decrease) and the days since the inclusion to the last days of follow-up within the first week after VA-ECMO implantation

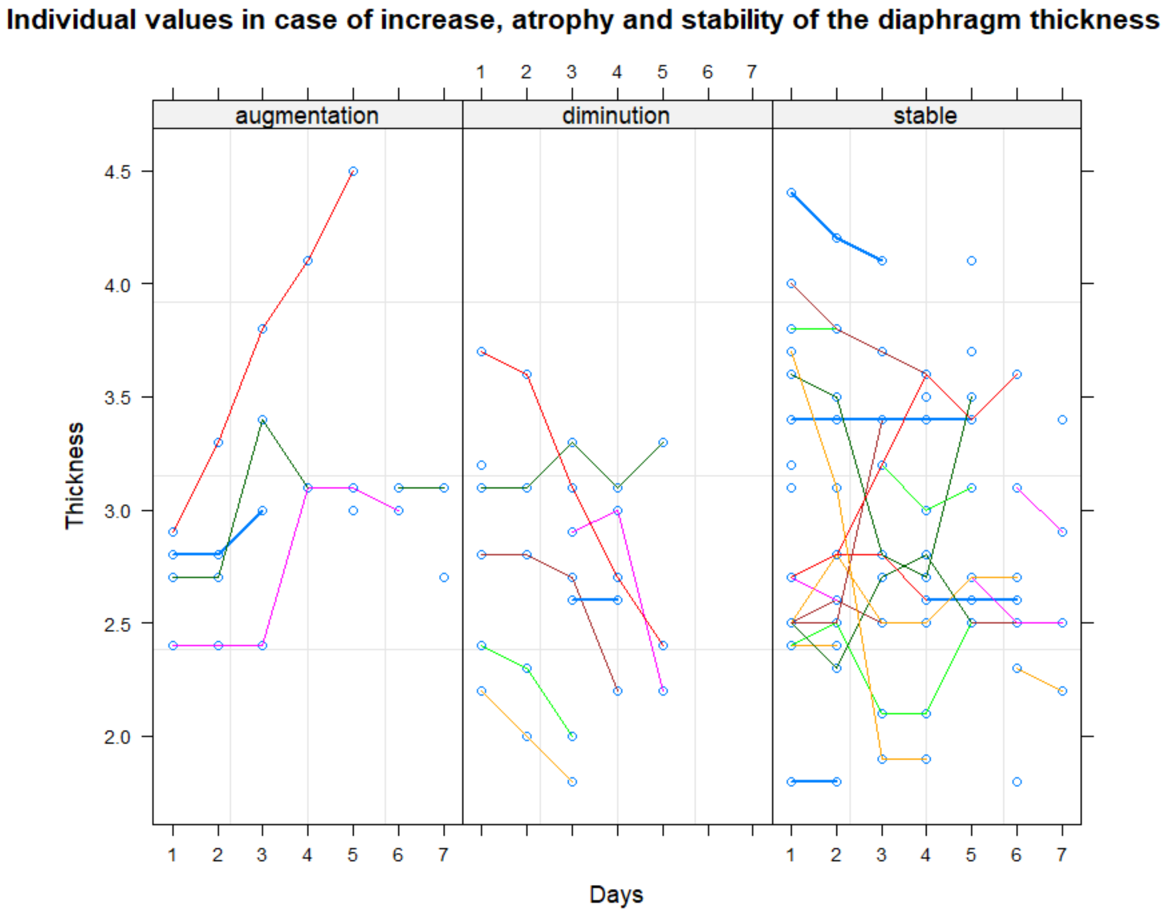

Figure S3: Graphic representations of repeated measure correlation

Observations from the same participant are given the same color, with corresponding lines to show the repeated measure correlation fit for each participant. A represents the repeated measure correlation fit for each participant of thickness at end-expiration and pH. B represents the repeated measure correlation fit for each participant of thickness at end-expiration and sweep gas flow. C the repeated measure correlation fit for each participant of thickness at end-expiration and insulin daily doses.

A

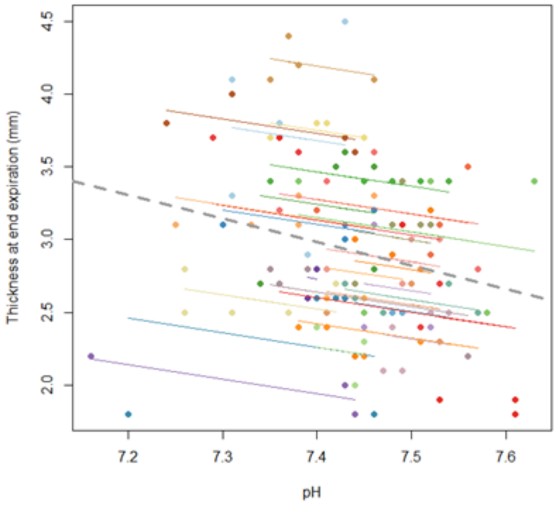

Thickness at end expiration (mm)
4.5
4.0
3.5
3.0
2.5
2.0
7.2
7.3
7.4
7.5
7.6
pH

B

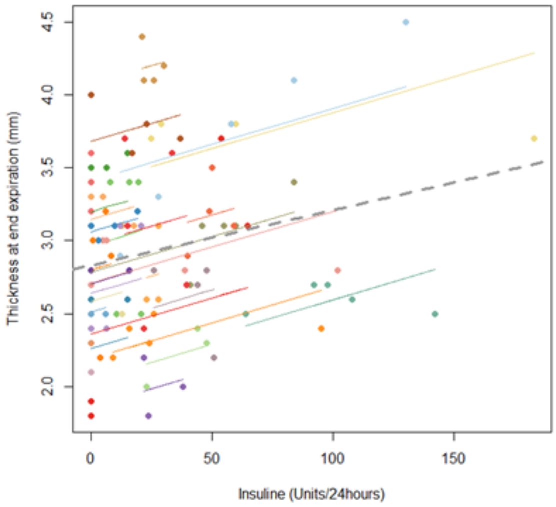

Thickness at end expiration (mm)
4.5
4.0
3.5
3.0
2.5
2.0
0
50
100
150
Insuline (Units/24hours)

C

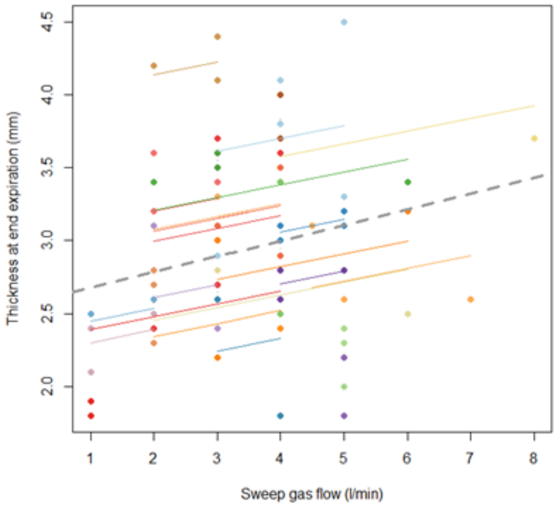

Thickness at end expiration (mm)
4.5
4.0
3.5
3.0
2.5
2.0
1
2
3
4
5
6
7
8
Sweep gas flow (l/min)

Figure S4: pH values in VA-ECMO in intubated or extubated patients by day three

The pH values were averaged over the three first days of the study period. Alkalosis was more frequent in extubated patients (7.48 [7.46, 7.49] in extubated patients versus 7.38 [7.36, 7.43)] in intubated patients; p-value = 0.006),

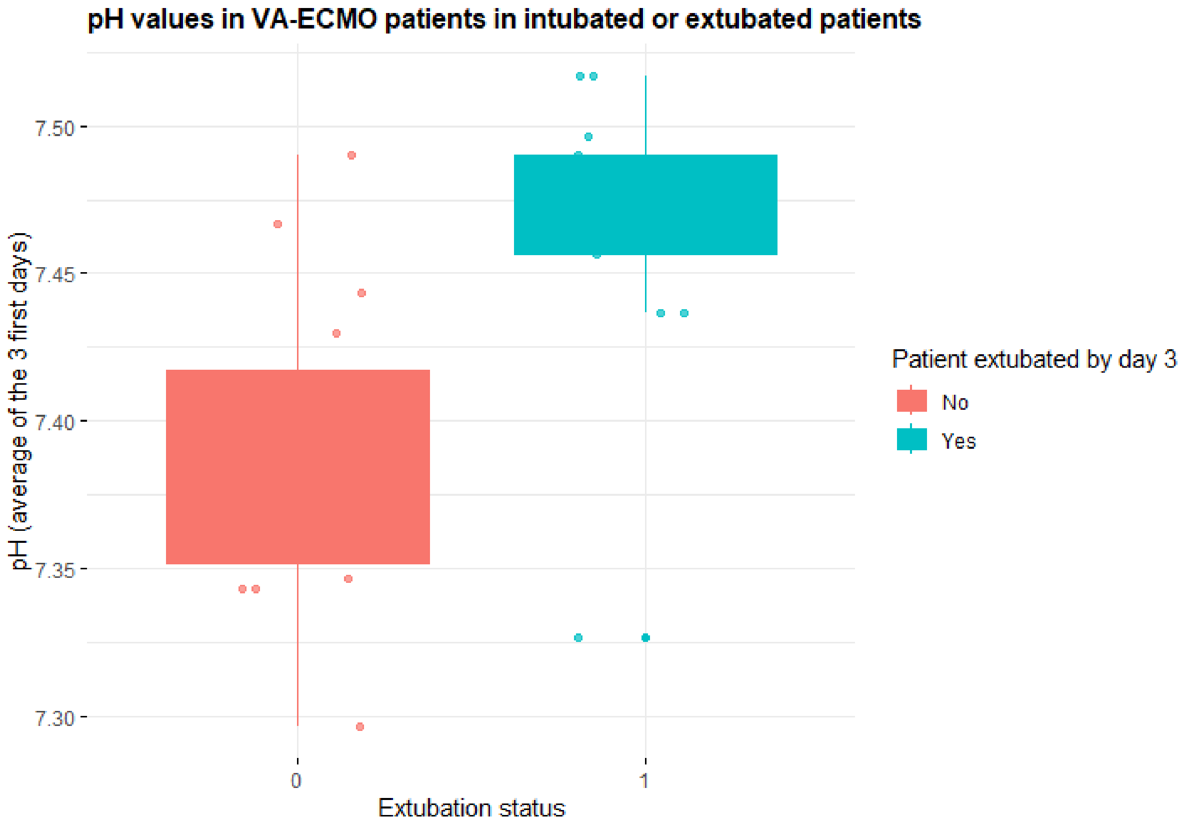

Figure S5: Sweep gas flow values in VA-ECMO in intubated or extubated patients by day three

The sweep gas flow values were averaged over the three first days of the study period. Extubated patients had a significantly lower sweep gas flow (4 l/min [4, 5] intubated patient versus 3 l/min [2,3] in extubated patients, p-value=0.002),

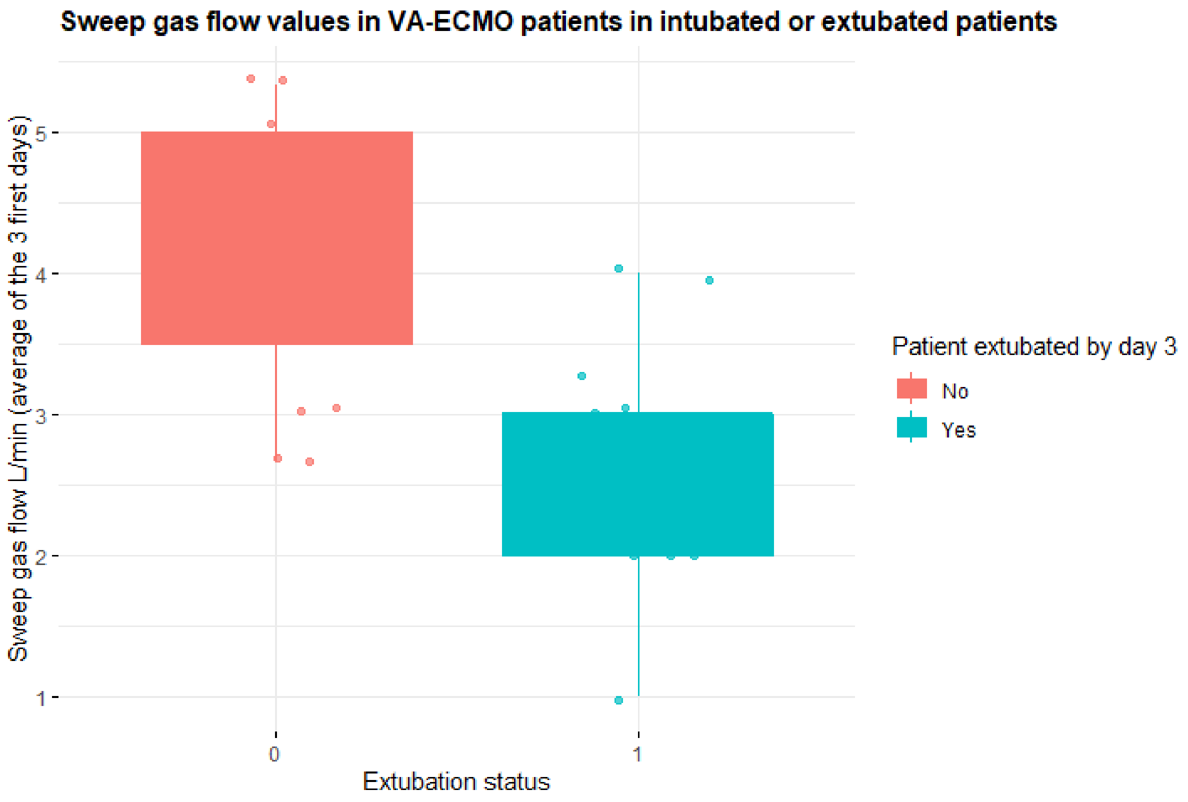

Figure S6: $PaCO_2$ (mmHg) values in intubated or extubated patients by day three

The $PaCO_2$ (mmHg) values were averaged over the three first days of the study period. $PaCO_2$ was lower in extubated patients (42 mmHg [39, 44] in intubated versus 38 mmHg [38, 39] in extubated; p-value = 0.024)

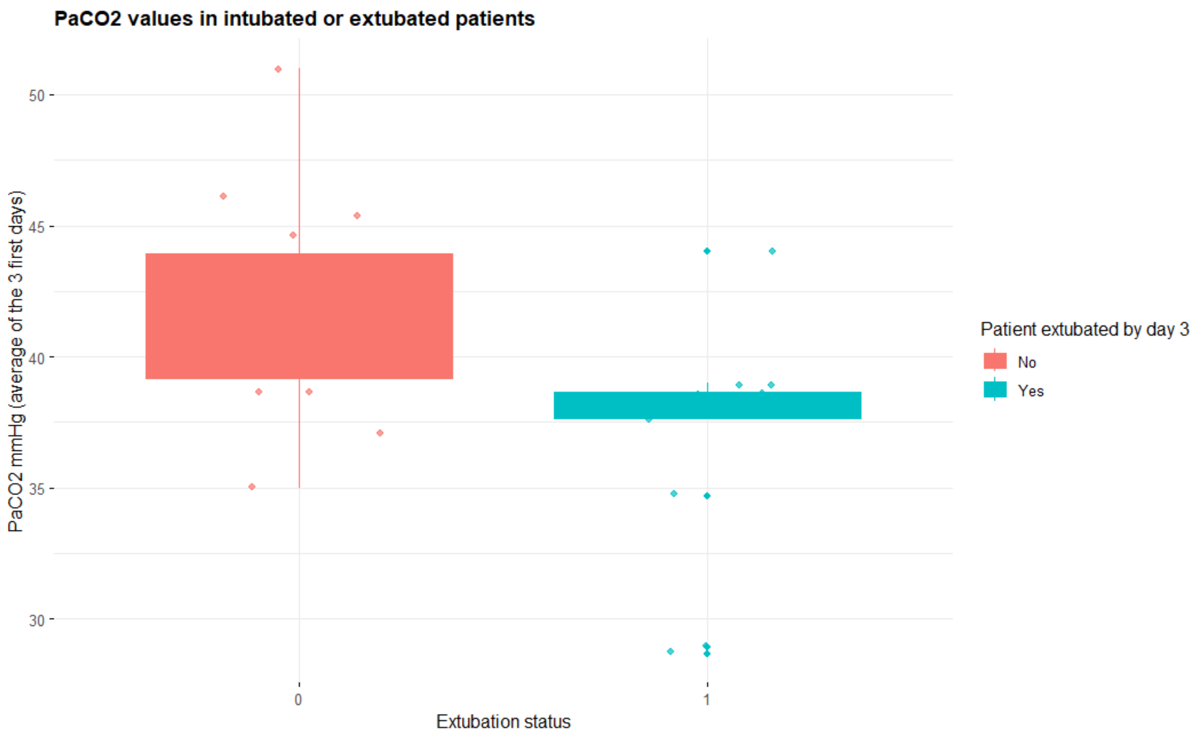

Figure S7: VA-ECMO flow values in intubated or extubated patients by day three

The VA-ECMO flow (L/min) values were averaged over the three first days of the study period.

The VA-ECMO output is a major contributor to PaO_2 and showed no significant difference at 4 l/min [3, 5] in intubated patients versus 3 l/min [3, 4] (p-value=0.2)

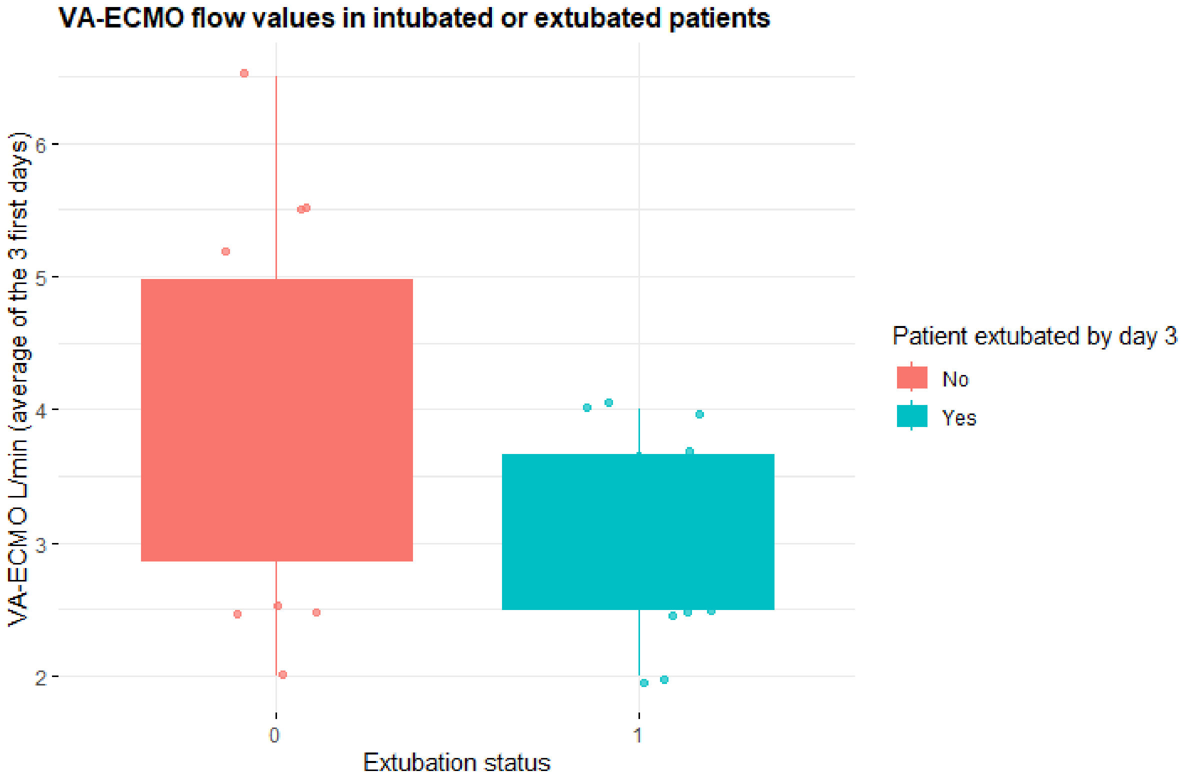

Figure S8: pH evolution according to the sweep gas flow according to the three groups of thickness classification.

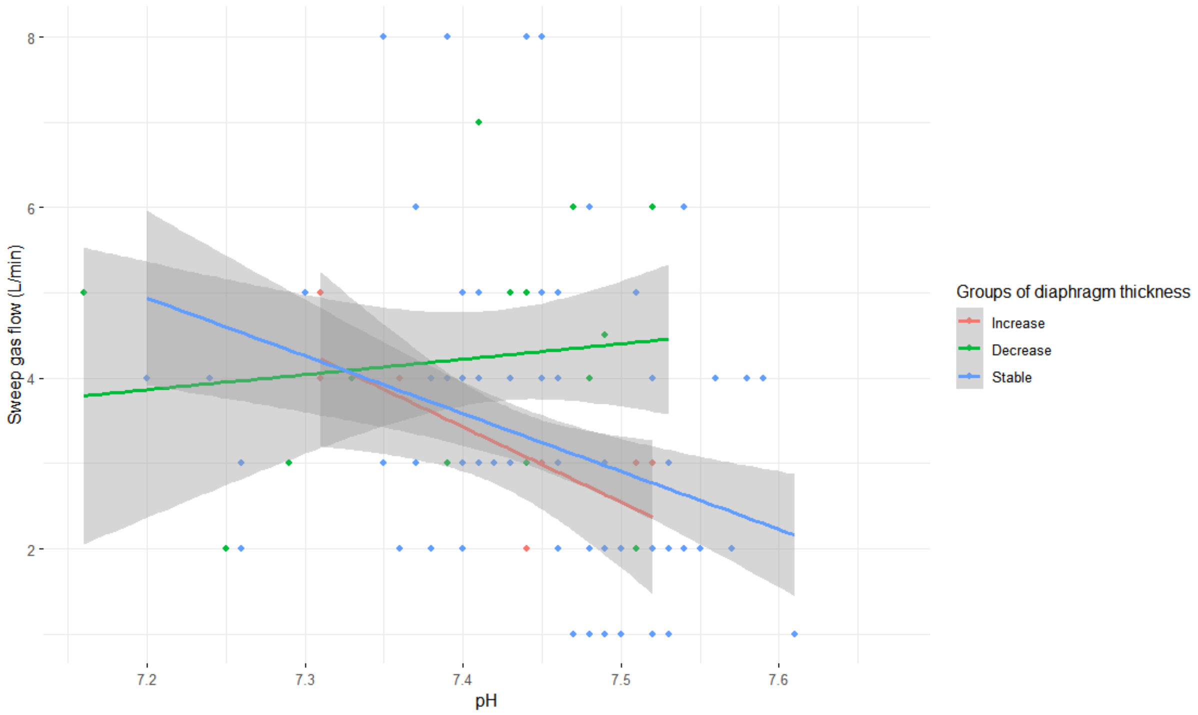

Figure S9: Visual abstract

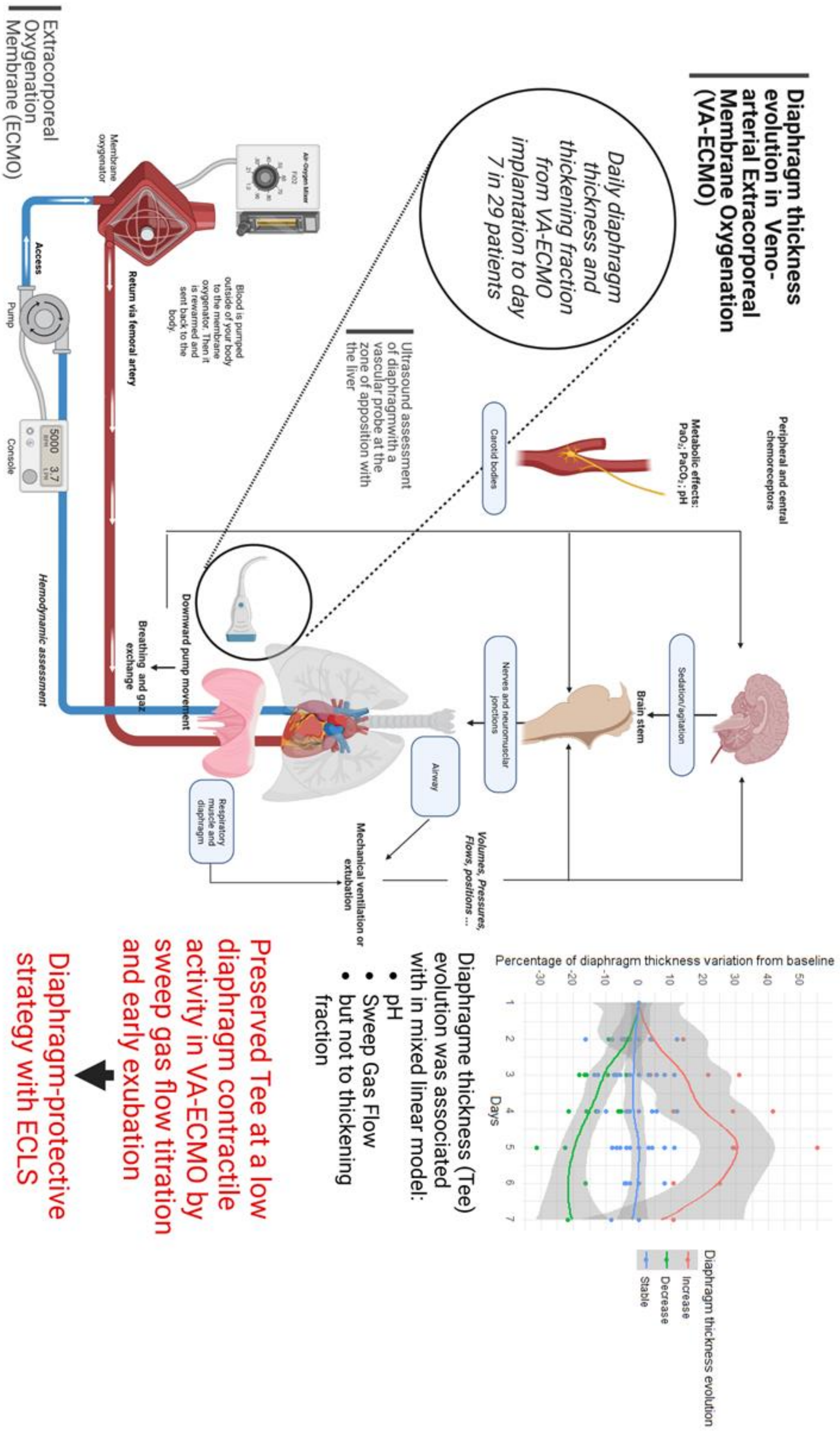

Supplement: Supplementary file 1 — Additional file 1:Table 1. Included cardiopathy, type of surgery procedures and septic status in the studied population. Table 2. Patients characteristics according to their liberation from mechanical ventilation status at day 3. Table 3. Patients characteristics according to their diaphragm evolution status at the end of patient follow-up. Table 4. Diaphragm thickness at end expiration evolution in cardiogenic shock treated with VA-ECMO: mixed-linear model and sensitivity analysis. Table 5. Diaphragm thickening fraction (dTF %) evolution in patient extubated before day 4 or not. Table 6. Patients characteristics according to the diaphragm contractile activity. Table 7. Diaphragm thickening fraction evolution in cardiogenic shock treated with VA-ECMO: calculation of a mixed-effect linear model (MLM) of diaphragm thickness over time, with subjects treated as random effects. Table 8. Patients outcome according to their liberation from mechanical ventilation status at day 3 Table 9. Outcome of the studied population according to the contractile activity status. Table 10. Univariate analysis of death at 60 days. Logistic regression model of the risk of death at 60 days adjusted for age, body mass index, cardiac arrest, SAPS2 and the presence of diaphragm atrophy by day 7. Table 11. Characteristics of patients deceased during the 60 days follow-up after the implantation of a VA-ECMO. Figure 1. Study flowchart. Figure 2. Graphic representations of the individual values of thickness evolution. Figure 3. Graphic representations of repeated measure correlation. Figure 4. pH values in VA-ECMO in intubated or extubated patients by day three. Figure 5.Sweep gas flow values in VA-ECMO in intubated or extubated patients by day three. Figure 6. PaCO2 (mmHg) values in intubated or extubated patients by day three. Figure 7.VA-ECMO flow values in intubated or extubated patients by day three. Figure 8.pH evolution according the sweep gas flow according the three groups of thickness cl [file 13613_2024_1264_MOESM1_ESM.pdf]
